# Resting-State Theta and Alpha Oscillations in Amputation and Phantom Limb Pain: A Pre-Registered High-Density EEG Study

**DOI:** 10.1007/s10548-026-01210-w

**Published:** 2026-05-22

**Authors:** Malin Ramne, Eva Lendaro

**Affiliations:** 1 Department of Electrical Engineering, Chalmers University of Technology, Gothenburg, Sweden; 2 Department of Brain and Cognitive Sciences, MIT, Cambridge, USA

**Keywords:** Phantom limb pain, Amputation, Resting-state, EEG, Electroencephalography, BIDS, Theta, Alpha oscillations, Peak alpha frequency, Chronic pain, Thalamocortical dysrhythmia, Alpha slowing

## Abstract

Phantom limb pain (PLP) affects a substantial portion of individuals with limb amputation, yet its neural mechanisms remain poorly understood. While neuroimaging studies have predominantly employed functional magnetic resonance imaging and magnetoencephalography, resting-state EEG could offer complementary insight into intrinsic brain oscillations but remains underexplored in this context. More generally, neuropathic pain has been associated with increased theta-band power and slowing of peak alpha frequency but whether these patterns extend to PLP is unknown. Here, we conducted a pre-registered, cross-sectional investigation of high-density resting-state EEG (58 channels) in 19 intact controls, 6 amputees without PLP, and 13 amputees with PLP. We employed mixed-effects models with bootstrap inference, multiple sensitivity analyses, and exploratory cluster-based permutation testing. Across primary and sensitivity analyses, we found no evidence for a robust association between PLP and either theta power or peak alpha frequency. Amputation-related differences in the alpha band reached statistical significance in some analyses, suggesting possible alpha-band alterations associated with limb loss rather than pain per se. Exploratory analyses showed a positive association between one measure of peak alpha frequency and pain intensity. However, inconsistent replication across spectral measures and tests indicates that these amputation and pain intensity-related findings should be interpreted with caution. For instance, results differed depending on the metric used (spectral maximum vs. center of gravity), highlighting the sensitivity of peak alpha frequency analyses to methodological choices. Together, these results suggest that resting-state EEG markers commonly reported in chronic pain do not straightforwardly generalize to PLP.

**Supplementary Information:**

The online version contains supplementary material available at 10.1007/s10548-026-01210-w.

## Introduction

Phantom limb pain (PLP) is a condition where pain is perceived as arising from a missing limb. Despite the high prevalence of PLP among individuals with amputation (Limakatso et al. 2020), the underlying mechanisms of this painful condition are poorly understood (Schone et al. 2022). A considerable body of neuroimaging studies on PLP have investigated changes in neural activation in somatosensory and motor cortices in response to imagined or executed movement or tactile stimulation (Andoh et al. 2020; Birbaumer et al. 1997; Flor et al. 1995; Foell et al. 2014; Grüsser et al. 2001; Huse et al. 2001; Karl et al. 2001; Kikkert et al. 2018; Knecht et al. 1995; Lotze et al. 2001; MacIver et al. 2008; Makin et al. 2013, 2015a, b; Raffin et al. 2016). Studies have additionally investigated structural and network-level changes in functional connectivity (Makin et al. 2013, 2015a, b). Comparatively little attention has been given to neural oscillations in the context of phantom limb pain. Resting-state EEG captures spontaneous brain rhythms independent of task demands, offering a non-invasive, accessible window into intrinsic neural dynamics, easy to use in both clinical and research contexts.

Neural oscillations reflect the synchronized activity of neuronal populations and are critical for coordinating information processing across distributed brain networks (Gyorgy and Andreas 2004). In the context of pain, oscillatory changes could be a sign of disrupted sensory processing, altered attention allocation, or maladaptive learning processes that contribute to chronic pain maintenance (Ploner et al. 2017). Therefore, investigating resting-state oscillatory patterns can provide additional and complementary insight into the neural mechanisms sustaining chronic pain.

Prior research investigating neuro-oscillatory neural correlates of chronic pain has produced heterogeneous results, with few consistent patterns emerging across studies. Systematic reviews of resting-state EEG studies suggest that chronic and neuropathic pain is associated with increased theta power, alongside some evidence of a slowing of the peak alpha frequency (Dos Santos Pinheiro et al. 2016; Mussigmann et al. 2022; Zebhauser et al. 2023). Other findings with tentative evidence include elevated gamma power and enhanced theta-band connectivity in chronic pain (Zebhauser et al. 2023), and decreased power within the high-alpha to low-beta range (10–20 Hz) and increased peak frequencies in the beta range in neuropathic pain (Mussigmann et al. 2022).

The only prior study we could find on resting state EEG and phantom limb pain demonstrated a decrease in beta and gamma activity, as well as decrease in theta and delta power in posterior temporal regions during eyes-open, with decreased delta power on the right side during eyes-closed (Bagheri et al. 2023). We summarize these findings in Table 1.

**Table 1:** Summary of neuro-oscillatory biomarkers of chronic, neuropathic and phantom limb pain as identified in systematic reviews and studies. Symbols: $\:\uparrow\:$ increase in power; $\:\downarrow\:$ decrease in power; $\:\leftarrow\:$ slowing (left-shift) of peak frequency; $\:\to\:$ quickening (right-shift) of peak frequency

Ref	Delta power	Theta power	Alpha		Beta		Gamma power
			Power	Peak frequency	Power	Peak frequency	
Systematic review – chronic pain (Dos Santos Pinheiro et al. 2016)	$\:\uparrow\:$	$\:\uparrow\:$	$\:\uparrow\:$	$\:\leftarrow\:$	$\:\uparrow\:$		
Systematic review – neuropathic pain (Mussigmann et al. 2022)		$\:\uparrow\:$	$\:\downarrow\:$	$\:\leftarrow\:$	Low $\:\downarrow\:$ High $\:\uparrow\:$	$\:\to\:$	
Systematic review – chronic pain (Zebhauser et al. 2023)		$\:\uparrow\:$		$\:\leftarrow\:$	$\:\uparrow\:$		$\:\uparrow\:$
Study on PLP (Bagheri et al. 2023)	$\:\downarrow\:$ for EO and EC (in different regions)	$\:\downarrow\:$ for EO			$\:\downarrow\:$ for EO		$\:\downarrow\:$ for EO

As illustrated in the table above, the existing findings are heterogeneous and do not converge on a single consistent oscillatory signature of chronic pain. Nevertheless, two patterns appear repeatedly across studies: increased theta-band power and a slowing of peak alpha frequency. These oscillatory changes have been attributed to several mechanisms. One influential account is the concept of *thalamocortical dysrhythmia*, which proposes that partial sensory deafferentation alters the firing patterns of thalamic relay neurons, shifting thalamocortical network activity from the normal alpha range toward slower theta-frequency oscillations *(*Llinás et al. 1999). In this model, reduced thalamic input destabilizes thalamocortical circuits and weakens inhibitory gating, leading to increased low-frequency activity and abnormal cortical excitability that may contribute to persistent percepts such as chronic pain. Because limb amputation results in a loss of peripheral sensory input, similar thalamocortical mechanisms might plausibly be engaged in PLP. Within this framework, PLP could therefore be associated with increased theta activity and a slowing of peak alpha frequency, motivating the present investigation of these oscillatory patterns.

To investigate if these oscillatory patterns replicate in PLP, we analyze resting state high-resolution EEG data from three different groups: amputees with PLP (AmpPLP), amputees without PLP (AmpNoPLP) and individuals without amputation (IC, intact controls). Specifically, our primary research questions are:

RQ1: Is resting state EEG theta band power increased in individuals with phantom limb pain?

RQ2: Is there a slowing of resting state EEG peak alpha frequency in individuals with phantom limb pain?

We also perform exploratory analysis of additional potential differences between the groups in our dataset.

## Methods

### Participants

The dataset contains data from three groups: 13 amputees with PLP (AmpPLP), 6 amputees without PLP (AmpNoPLP), and 19 controls with intact limbs (IC). Data collection was approved by the Swedish Ethical Review Authority (Dnr 041 − 17, T652-17) and all participants provided written informed consent.

### Data Acquisition

During data acquisition sessions, participants first completed clinical and demographic questionnaires. For this study, the following demographic variables are used: age, sex, side and level of amputation. Participants with PLP also completed a questionnaire to quantify and characterize their pain. The list of questions and responses are included in the data repository. For the purposes of this analysis, we used the numeric rate scale (NRS) score evaluated at the time of the recording.

At each recording session EEG data was collected for two resting state conditions: eyes closed and eyes open, however we only analyzed the eyes-closed data in this study. The participants were instructed to stay relaxed and awake during the recordings. Data was collected for 7 min with the participants sitting comfortably in a chair in a quiet room. In the eyes open condition participants were instructed to rest their eyes on a white cross on a black background displayed in front of them on a screen. At most sessions two recordings were collected for each condition (total 4 recordings per session) with approximately 4-minute breaks between recordings. Some participants with PLP participated at multiple recording sessions to capture possible differences related to varying levels of pain. Furthermore, some of these participants took part in a clinical trial evaluating treatments for PLP (Lendaro et al. 2025).

EEG data was collected with 64 or 128 electrodes placed according to the international 10/10 system using the g.GAMMAcap2 from g.tec medical engineering GmbH and with a g.HIamp amplifier from the same company. During data analysis only the 58 common electrodes between the two montages are used. Data was sampled at 2400 Hz with no real-time processing or filtering.

### Data Preprocessing

EEG recordings were preprocessed using the DISCOVER-EEG pipeline for biomarker discovery in resting state EEG data (Gil Ávila et al. 2023). Prior to applying the DISCOVER-EEG pre-processing pipeline recordings were reduced to the 58 electrodes that are common between the two montages (64 and 128 channels) and the first and last 30 s of each recording were truncated. Then, in the DISCOVER-EEG pipeline, line noise was removed, the data was high-passed with transition band of 0.25 to 0.75 Hz, and artifactual channels were automatically detected and removed if they were flat for more than 5 s, had a noise-to-signal ratio larger than 4, or were poorly correlated with other channels (< 80% of the time). Data were re-referenced to the common average and subjected to independent component analysis. Components identified as muscle or eye artifacts (probability > 80%) were subtracted from the data. Removed channels were then interpolated using spherical splines to maintain consistent dimensionality across participants. To address transient artifacts, artifact subspace reconstruction was applied to remove segments with variance 20 times higher than a clean calibration period. Finally, the continuous data were segmented into 5-second epochs with 50% overlap, and any epochs containing remaining discontinuities were rejected. The DISCOVER-EEG pre-processing pipeline was then run with mostly default settings except for the following modifications:

- Epoch length 5 s instead of 2 s (for better frequency resolution).
- 5 ICA repetitions instead of 10 (to speed up pre-processing),
- ZapLine Plus instead of CleanLine for line noise removal (due to better perceived performance of ZapLine Plus) (Klug and Kloosterman 2022).

### Feature Extraction

Frequency analysis was also performed using the DISCOVER-EEG pipeline (Gil Ávila et al. 2023). Continuous EEG data were segmented into 5-second epochs with a 50% overlap. This epoch length was chosen specifically to enhance frequency resolution compared to the pipeline’s 2-second default. Power spectral density was computed between 1 and 100 Hz using Slepian multitapers with +/−1 Hz frequency smoothing. The resulting frequency resolution was 0.2 Hz. Power spectra were computed independently for every epoch and then averaged across epochs to form a single power spectrum per channel. Finally, a grand average power spectrum per trial was computed by averaging across the 58 channels.

Peak alpha frequency was identified within the 8–12.9 Hz range using two distinct criteria: (1) **PAF-Max**, defined as the frequency of the local maximum (highest power) within the alpha band, and (2) **PAF-CoG**, calculated as the center of gravity (the weighted average of frequencies by their power) to provide a more robust estimate in cases of multiple or broad peaks. Theta band power was calculated by averaging the power spectral density across the 4–7.9 Hz range.

### Data Analysis and Statistical Comparisons

The data analysis plan has been pre-registered at: https://osf.io/rw2ma. To prevent bias during development of analysis scripts, all group labels were randomly permuted prior to running any statistical models. Specifically, the labels for Amputee and PLP (nested within Amputees), were randomly reassigned while preserving the original group size proportions. Analyses and scripts were finalized under these blinded labels. In this study we only analyzed data from the eyes-closed recordings.

#### RQ1 – Theta Power

To investigate potential differences in theta band power used a linear mixed-effects model with random intercepts for subjects to account for within-subject correlation and varying numbers of trials. The dependent variable was the log-transformed per-trial theta power averaged across the whole frequency band (4–7.9 Hz). Fixed effects included Amputee (0 = intact control, 1 = amputee), PLP (1 = PLP group, 0 = otherwise), Age, and Sex, resulting in the following model formula:

$$\begin{aligned}\:{\stackrel{-}{\theta\:}}_{ij}&={\beta\:}_{0}+{\beta\:}_{\mathrm{Amp}}{\mathrm{Amputee}}_{i}+{\beta\:}_{\mathrm{PLP}}{\mathrm{PLP}}_{i}\\&\quad+{\beta\:}_{\mathrm{Age}}{\mathrm{Age}}_{i}+{\beta\:}_{\mathrm{Sex}}{\mathrm{Sex}}_{i}+{{u}_{i}+\:\epsilon\:}_{ij}\:\end{aligned}$$

where $\:{u}_{i}$​ is a random intercept for subject $\:i$. The coefficient $\:{\beta\:}_{\mathrm{PLP}}$ represents the PLP-specific effect and serves as the primary confirmatory test. The coefficient $\:{\beta\:}_{\mathrm{Amp}}$ represents the amputation-specific effect.

A *subject-level linear regression model* using per-subject average theta power was used to verify consistency of direction and effect size of the mixed-effects models in the main analysis. Unadjusted pairwise t-tests comparing AmpPLP vs. AmpNoPLP and AmpNoPLP vs. IC are also reported for transparency.

Considering the limited sample size of our dataset 95% confidence intervals (CIs) for fixed effects were estimated using bootstrapping (resampling subjects with replacement). For mixed models, bootstrapping was performed at the subject level to preserve the within-subject correlation structure.

Because the sample size is limited, and normality assumptions may be fragile, statistical inference is based on bootstrap CIs. If the bootstrap and model-based (asymptotic) CIs disagree, interpretation rely on the bootstrap CI, as it makes fewer distributional assumptions. We still report model-based p-values for transparency, but they do not determine significance.

To explore finer frequency-specific effects within the theta band we performed a secondary analysis using cluster-based permutation test (CBPT) across frequencies within the theta range. This analysis is adapted from the validation analysis from Fig. 4. in the DISCOVER-EEG publication (Gil Ávila et al. 2023) which uses the FieldTrip toolbox (Oostenveld et al. 2011). Our adaptation of the analysis involves three significant changes:

- we limit our analysis only to the theta band range (4–7.9 Hz),
- we use *independent* samples CBPT since we are comparing between distinct groups instead of between conditions within a group,
- we use one-tailed t-test since our hypothesis is directional.

The analysis uses the Fieldtrip toolbox in Matlab. To our knowledge the toolbox currently does not support multiple independent variables in the CBPT. Therefore, we use pairwise *independent-samples regression t-statistics* on the following comparisons:

i. AmpPLP vs. NoAmpPLP for PLP specific differences,
ii. NoAmpPLP vs. IC for amputation specific differences,
iii. If comparison ii. shows no significant differences (no clusters with *p* < 0.05): AmpPLP vs. [NoAmpPLP + IC]. Pooling increases sample size and statistical power for detecting PLP-related neural differences but may introduce potential amputation effects. Therefore, this analysis is only conducted if the secondary analysis indicates that such effects are negligible.

We infer statistical significance if the p-values from the cluster-based permutation test are lower than 0.05 for any clusters.

#### RQ2 – Peak Alpha Frequency

To investigate potential differences in peak alpha frequency trial-level linear mixed-effects models were fitted using the same fixed and random effects structure as for RQ1. We fit one model per peak alpha frequency measure (PAF-Max and PAF-CoG). The coefficient $\:{\beta\:}_{\mathrm{PLP}}$ of the PAF-Max model is the primary confirmatory test for PLP-specific differences, as this measure is most commonly used in prior studies. The PAF-CoG is also a recognized but somewhat less utilized measure of peak alpha frequency. Therefore, we treat the $\:{\beta\:}_{\mathrm{PLP}}$ coefficient of the PAF-CoG model as secondary. Similarly, the $\:{\beta\:}_{\mathrm{Amp}}$ coefficients are examined for amputation-specific effects.

Robustness check and inference criteria follow the same procedure as for RQ1.

#### Deviations from Pre-registered Analysis

We used 5000 bootstrapping samples instead of 2000 for the CIs, for improved robustness of the estimates.

#### Exploratory Analyses

To further explore potential differences in power we expand the CBPT analysis from RQ1. In particular, we apply the analysis to the alpha band (8–13 Hz) to further investigate potential differences contributing to shifts in peak alpha frequency, and to the full power spectrum (1–100 Hz) to investigate any other potential differences in power. Additionally, we extend the analysis to frequency-by-channels space to identify spatial location of differences. For this analysis we will use two-tailed t-test since we do not have a specific hypothesis about the direction of differences.

As an additional exploratory analysis, we examined whether theta power or peak alpha frequency were associated with pain intensity within the group of amputees reporting PLP. Pain intensity ratings, available for a subset of recordings, were entered as a continuous predictor in a trial-level linear mixed-effects model restricted to PLP subjects, with random intercepts for subjects and adjustment for age and sex. Analyses were limited only to sessions with available pain ratings and were not part of the preregistered primary hypotheses.

### Code and Data Availability

The data is available open access in BIDS-EEG format at: https://openneuro.org/datasets/ds006921.

Code for the statistical analysis is available at: 10.5281/zenodo.17977343.

## Results

### Demographic and Clinical Variables

Overall demographics pertaining to age, sex, amputation side and limb are presented in Table 2. The groups are not balanced regarding size, sex or age, with the IC group being larger and skewing younger and more female than the two groups of amputees. While these discrepancies are adjusted for in the statistical analysis, they may affect the statistical power of the analyses.

**Table 2:** Demographics of the subjects included in the dataset

	*n*	Mean	Range	Sex ratio M: F	Amp side ratio L: *R*	Amp limb ratio UL: LL
IC	19	25.3	(22–28)	6:13	–	–
AmpNoPLP	6	49.7	(39–77)	5:1	3:3	4:2
AmpPLP	13	48.8	(27–66)	9:4	2:9 + 2 bilateral	7:5 + 1 quadruple

### Research Question 1: Theta Power

As the primary analysis we fitted a linear mixed-effects model predicting per-trial theta power from PLP, Amputee, Age, and Sex, with a random intercept for subject. Theta power did not differ significantly between AmpPLP and AmpNoPLP groups (fixed effect $\beta_{\mathrm{PLP}}$ = 0.466, 95% bootstrapped CI = [-0.219, 1.120]) or between AmpNoPLP and IC (fixed effect $\beta_{\mathrm{Amp}}$ = 0.081 with 95% bootstrapped CI = [-0.963, 0.885]). Sex was a significant predictor of theta power, with males showing lower theta power than females ( $\beta_{\mathrm{Sex}}$ = -0.734, 95% bootstrapped CI = [-1.181, -0.233]). This effect was not part of our pre-registered hypotheses and is therefore interpreted as a covariate adjustment outcome. Age showed no association with theta power.

Model coefficients, 95% bootstrapped CIs, 95% asymptotic CIs and p-values are summarized in Table 3.

**Table 3:** Estimated model coefficients, 95% bootstrapped CIs, 95% asymptotic CIs and p-values for the linear mixed-effects model predicting per-trial theta power

Coefficient	Estimated effect	Bootstrapped CI	Asymptotic CI	*p*-value
$\:{\beta\:}_{\mathrm{PLP}}$	0.466	[-0.253, 1.104]	[-0.276, 1.207]	0.2147
$\:{\beta\:}_{\mathrm{Amp}}$	0.081	[-0.954, 0.881]	[-0.937, 1.100]	0.8741
$\:{\beta\:}_{\mathrm{Age}}$	0.013	[-0.334, 0.659]	[-0.379, 0.404]	0.9490
$\:{\beta\:}_{\mathrm{Sex}}$	-0.734	[-1.181, -0.233]	[-1.276, -0.191]	< 0.05

The marginal R² was 0.271, indicating that the fixed effects (Amputation, PLP, Age, Sex) accounted for 27.1% of the variance after accounting for subject-level random effects (effect size Cohen’s f^2^ = 0.372 and approximate Cohen’s d ≈ 1.22, provided for descriptive comparison only). The conditional R² was 0.883, reflecting the substantial contribution of individual differences captured by the random intercepts. Model diagnostics did not indicate violations severe enough to invalidate the resulting inferences, see Supplemental Material for details.

**Fig. 1:**
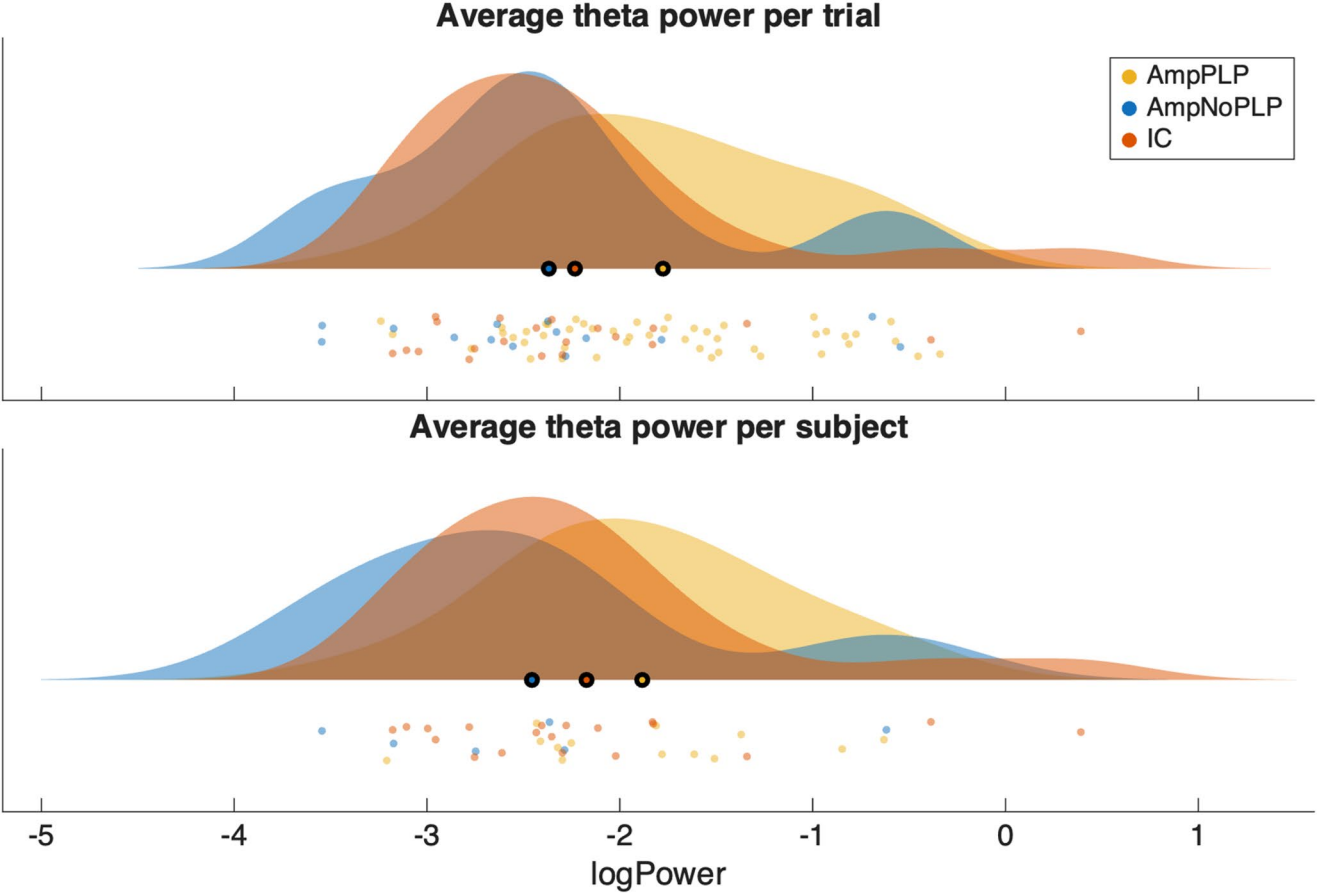
Raincloud plot of average theta power per trial (upper) and average per subject (lower) in the three different groups

As a robustness check we repeated the above analysis on per-subject averages of theta power using a linear regression model. This analysis was consistent with the mixed-effects model – no significant effects for PLP, amputation or age, while Sex remained a significant predictor of theta power ( $\beta_{\mathrm{Sex}}$ = -0.735, 95% bootstrapped CI = [-1.246, -0.188]). We also performed unadjusted pairwise t-tests comparing AmpPLP vs. AmpNoPLP (*p* = 0.0855, CI = [-0.124, Inf], right-tailed) and AmpNoPLP vs. IC (*p* = 0.5248, CI = [-1.190, 0.624], two-tailed). These tests were consistent with the main analysis indicating no significant effect of amputation or PLP on theta power. Figure 1 shows raincloud plots of the theta power per trial and per-subject average in the three different groups.

**Fig. 2:**
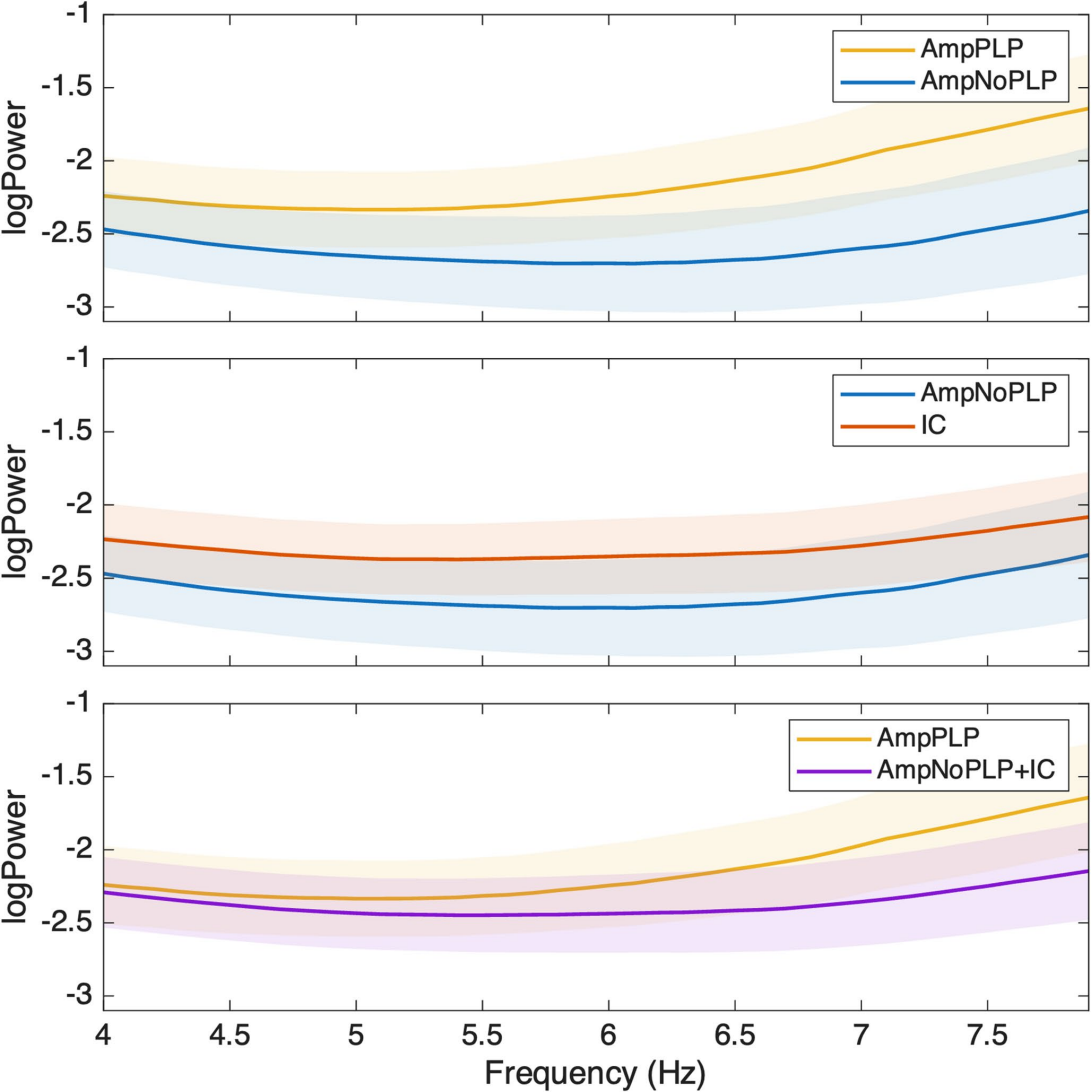
Power spectrum in the theta band for the three pairwise comparisons. No significant clusters were identified

Finally, we performed a one-tailed independent-samples cluster-based permutation test (CBPT) across the theta range (4–7.9 Hz) using subject-level averaged spectra. No significant clusters were detected for any of the pairwise comparisons, adjusted or unadjusted. Figure 2 shows the power spectrums in each comparison.

### Research Question 2: Peak Alpha Frequency (PAF)

Using the same fixed and random effects structure as for RQ1, the two different measures of peak alpha frequency (PAF-Max and PAF-CoG) were modeled at the trial level, with PAF-Max as the primary confirmatory test.

PAF-Max did not differ significantly between AmpPLP and AmpNoPLP groups (fixed effect $\beta_{\mathrm{PLP}}$ = 0.359, 95% bootstrapped CI = [-0.496, 0.965]). However, for Amputation the mixed-effects model of PAF-Max did indicate a statistically significant effect (fixed effect $\beta_{\mathrm{Amp}}$ = -1.227, 95% bootstrapped CI = [-2.391, -0.063]). Neither Sex nor Age showed associations with PAF-Max. See Table 4 for model coefficients, 95% bootstrapped CIs, 95% asymptotic CIs and p-values.

**Table 4:** Estimated model coefficients, 95% bootstrapped CIs, 95% asymptotic CIs and p-values for the linear mixed-effects model predicting per-trial PAF-Max

Coefficient	Estimated effect	Bootstrapped CI	Asymptotic CI	*p*-value
$\:{\beta\:}_{\mathrm{PLP}}$	0.359	[-0.496, 0.965]	[-0.423, 1.142]	0.3628
$\:{\beta\:}_{\mathrm{Amp}}$	-1.227	[-2.255, -0.037]	[-2.391, -0.063]	*p* < 0.05
$\:{\beta\:}_{\mathrm{Age}}$	0.393	[-0.341, 0.882]	[-0.015, 0.802]	0.0590
$\:{\beta\:}_{\mathrm{Sex}}$	0.171	[-0.461, 0.691]	[-0.426, 0.769]	0.5685

The mixed-effects model explained only a modest proportion of variance (marginal R² = 0.082; conditional R² = 0.164), suggesting that the fixed predictors exert limited predictive influence on APF-Max (effect size Cohen’s f^2^ = 0.089 and approximate Cohen’s d ≈ 0.6, provided for descriptive comparison only). See Supplemental Material for additional model diagnostics.

The Amputation effect was not reproduced in a subject-level linear model ( $\beta_{\mathrm{Amp}}$ = -0.911, 95% bootstrapped CI = [-2.383, 0.174]), in which all predictors were nonsignificant and the adjusted R² was negative, or in unadjusted pairwise t-tests (AmpPLP vs. AmpNoPLP: *p* = 0.4485, CI = [-0.829, Inf], right-tailed; AmpNoPLP vs. IC: *p* = 0.5899, CI = [-1.242, 0.724], two-tailed).

For the PAF-CoG measure, neither PLP (fixed effect $\beta_{\mathrm{PLP}}$ = 0.008, 95% bootstrapped CI = [-0.234, 0.251]) or Amputation (fixed effect $\beta_{\mathrm{Amp}}$ = -0.207, 95% bootstrapped CI = [-0.572, 0.186]) showed significant effects. Sex was a significant predictor of PAF-CoG ( $\beta_{\mathrm{Sex}}$= 0.333, 95% bootstrapped CI = [0.104, 0.566]). This effect was not part of our pre-registered hypotheses and is therefore interpreted as a covariate adjustment outcome. Age showed no association with APF-CoG.

Model coefficients, 95% bootstrapped CIs, 95% asymptotic CIs and p-values are summarized in Table 5.

**Table 5:** Estimated model coefficients, 95% bootstrapped CIs, 95% asymptotic CIs and p-values for the linear mixed-effects model predicting per-trial PAF-CoG

Coefficient	Estimated effect	Bootstrapped CI	Asymptotic CI	*p*-value
$\:{\beta\:}_{\mathrm{PLP}}$	0.008	[-0.234, 0.251]	[-0.369, 0.385]	0.9654
$\:{\beta\:}_{\mathrm{Amp}}$	-0.207	[-0.572, 0.186]	[-0.726, 0.312]	0.4300
$\:{\beta\:}_{\mathrm{Age}}$	-0.106	[-0.293, 0.049]	[-0.305, 0.093]	0.2920
$\:{\beta\:}_{\mathrm{Sex}}$	0.333	[0.104, 0.566]	[0.056, 0.609]	< 0.05

The marginal R² was 0.236, indicating that the fixed effects (Amputation, PLP, Age, Sex) accounted for 23.6% of the variance after accounting for subject-level random effects (effect size Cohen’s f^2^ = 0.309 and approximate Cohen’s d ≈ 1.11, provided for descriptive comparison only). The conditional R² was 0.861, reflecting the substantial contribution of individual differences captured by the random intercepts. Model diagnostics did not indicate violations severe enough to invalidate the resulting inferences, see Supplemental Material for details.

The subject level linear model was consistent with the mixed-effects model – no significant effects for PLP, amputation or age, while Sex remained a significant predictor of PAF-CoG ( $\beta_{\mathrm{Sex}}$ = 0.329, 95% bootstrapped CI = [0.046, 0.623] ). We also performed unadjusted pairwise t-tests comparing AmpPLP vs. AmpNoPLP (*p* = 0.5618, CI = [-0.345, Inf], right-tailed) and AmpNoPLP vs. IC (*p* = 0.3139, CI = [-0.670, 0.225], two-tailed). These tests were consistent with the main analysis indicating no significant effect of PLP or Amputation on PAF-CoG.

**Fig. 3:**
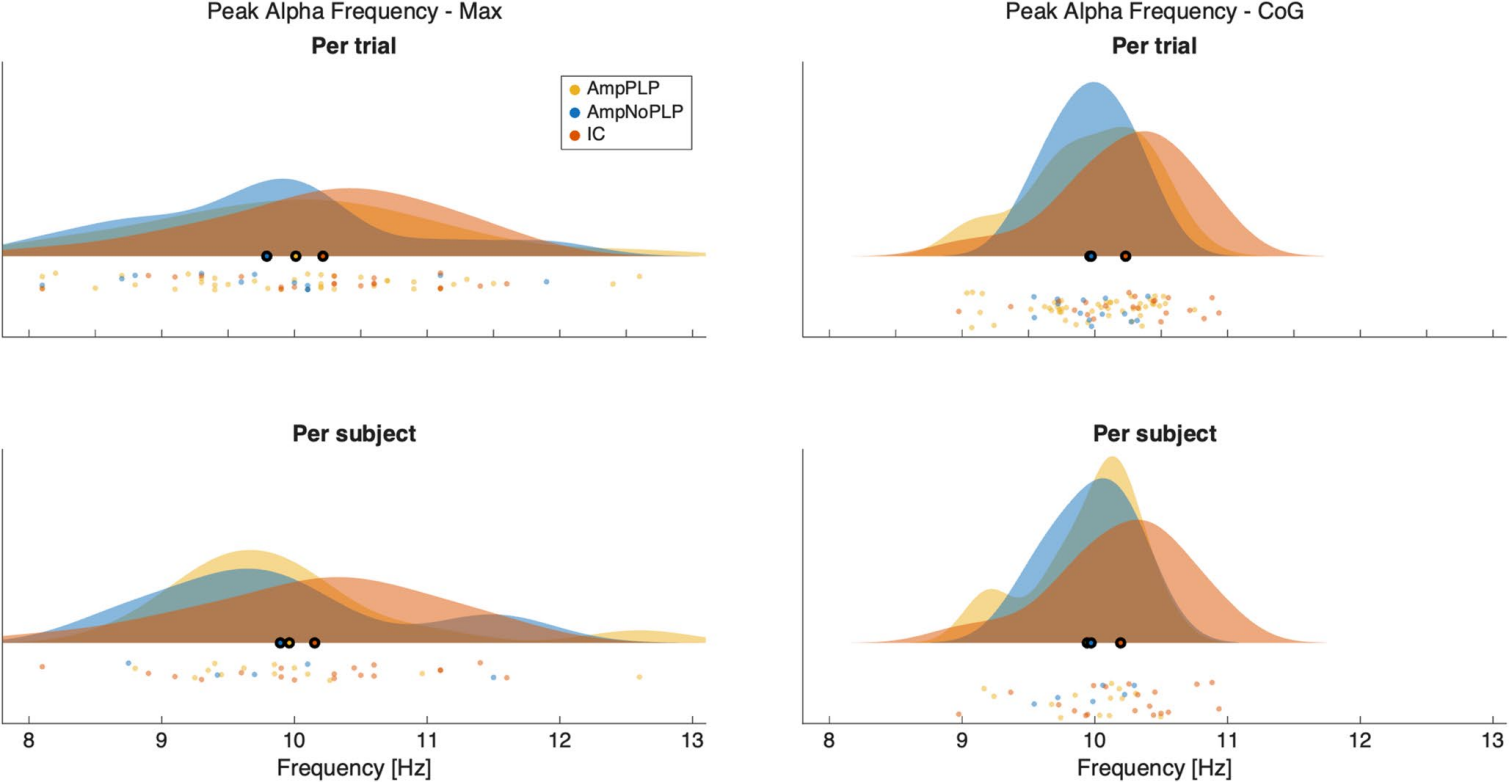
shows raincloud plots of the two peak alpha frequency measures per trial and per-subject average in the three different groups

Figure 3 raincloud plots of the two peak alpha frequency measures (PAF-Max and PAF-CoG) per trial and per-subject average in the three different groups.

### Exploratory Analyses

**Fig. 4:**
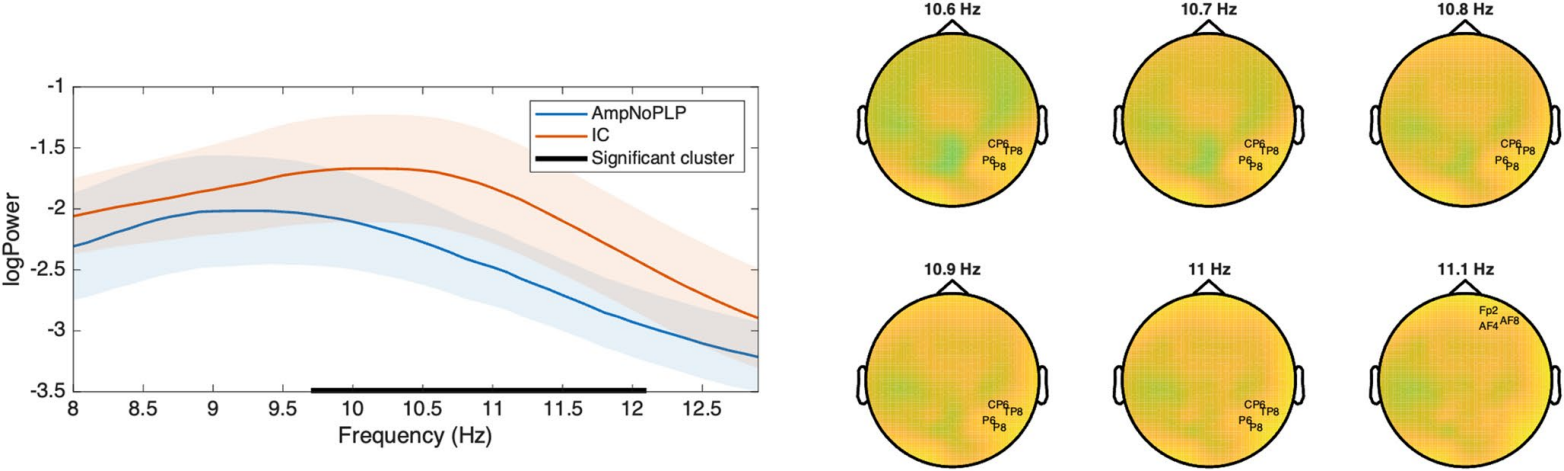
CBPT in the alpha band. The left panel shows the comparison of grand averages across channels, and the right panel shows the resulting significant clusters from the corresponding channel $\:\times\:$ frequency space analysis

To further investigate possible Amputation related differences related to the peak alpha frequency, we performed a similar CBPT as described above, but in the alpha band instead of the theta band. This analysis yielded a significant positive cluster in the frequency range 9.7–12.1 Hz, *p* < 0.05, in which AmpNoPLP have lower power than IC. When we expanded the CBPT to the channel-by-frequency space to explore possible regional specificity of the identified differences, two significant clusters were identified: 10.6–11 Hz for channels CP6, TP8, P6 and P8, and at 11.1 Hz for channels Fp2, AF8 and AF4. These results are visualized in Fig. 4.

The CBPT was also expanded to the full frequency range (0–100 Hz) to explore possible differences in power at other frequencies. However, no significant clusters were identified for group differences in any of the pairwise comparisons in this analysis.

In the exploratory analysis of pain intensity within the PLP group no significant associations were observed for theta power or PAF-Max. However, pain intensity did show a small but statistically significant positive association with PAF-CoG ( $\beta_{\mathrm{PainIntensity}}$= 0.05 Hz per pain unit, *p* < 0.05), indicating that higher reported pain intensity was associated with slightly faster PAF-CoG frequencies within individuals with PLP.

## Discussion

The present study investigated resting-state spectral EEG markers previously associated with chronic pain in the context of PLP. Across primary and sensitivity analyses, we found no robust evidence for PLP-specific alterations in either theta power or peak alpha frequency. An amputation-related shift in PAF-Max reached statistical significance in the primary mixed-effects model but was not reproduced in subject-level sensitivity analyses or in the complementary PAF-CoG measure.

Alpha oscillations arise from reciprocal thalamocortical interactions and intrinsic cortical circuits involving excitatory-inhibitory balance (Bollimunta et al. 2008; Hughes and Crunelli 2005; Lopes da Silva et al. 1980). Although early models emphasized superficial cortical generators, laminar recordings demonstrate that alpha activity spans multiple cortical layers and reflects coordinated interactions across thalamocortical and intracortical circuits (Haegens et al.,^ 2015^). Alpha power is maximal during eyes-closed rest and suppresses with eye opening (the “Berger effect”) reflecting shifts between cortical idling and active sensory processing states (Pfurtscheller et al. 1996). A slowing of peak alpha frequency has been attributed to several mechanisms but the most relevant here is what Llinás et al. (1999) termed *thalamocortical dysrhythmia (*Llinás et al. 1999). Thalamocortical dysrhythmia, originally proposed to explain tinnitus and neuropathic pain, proposes that loss of peripheral input triggers compensatory changes in thalamic neuron excitability and connectivity, leading to pathological low-frequency oscillations in the theta-alpha range that may generate abnormal percepts. The present results raise the possibility that alpha-band alterations following amputation may reflect consequences of sensory deafferentation that are not specific to the absence or presence of pain.

Exploratory CBPT analyses revealed alpha-band differences associated with amputation status. The frequency-by-channel analysis revealed alpha-band differences with a predominantly posterior distribution in the right hemisphere, independent of side of amputation. This posterior involvement is broadly consistent with cortical regions implicated in visuospatial attention and body representation. Speculatively, posterior alpha-band alterations may relate to changes in networks supporting body representation following limb loss. In particular, the right parietal cortex has been implicated in bodily awareness and body schema, as evidenced by its role in hemispatial neglect and related disorders (Heilman et al. 2000; Karnath et al. 2001; Vallar and Perani 1986).This raises the possibility that alpha-band alterations following amputation may relate to altered body schema representations following sensory deafferentation. A secondary exploratory analysis restricted to amputees with PLP showed a small positive association between pain intensity and PAF-CoG.

In summary, these findings may be reconciled by distinguishing between two separate processes following amputation: (1) deafferentation-related thalamocortical dysrhythmia affecting all amputees, and (2) variability in the persistence of the missing limb’s cortical representation, which may be influenced by pain outcomes. Amputation-related alpha-band alterations, particularly in right posterior regions implicated in body schema integration, may reflect the primary consequence of sensory deafferentation (akin to the body schema disruptions observed in hemispatial neglect following right parietal damage). However, within this context of disrupted input there may exist individual variation in the persistence of the cortical representation of the missing limb. Some studies suggest that PLP is associated with preserved cortical representations of the missing hand (Kikkert et al. 2018; Makin et al. 2013). In this framework, the positive association between pain intensity and PAF-CoG observed in our exploratory analysis may reflect the strength or integrity of the maintained representation: higher pain intensity may be the result of less thalamocortical dysrhythmia possibly due to ectopic peripheral discharge, resulting in faster alpha oscillations leading also to a more integrated body schema. Furthermore, this framework predicts that individuals without PLP and less vivid phantom sensations should exhibit more pronounced dysrhythmic patterns, and slower alpha frequencies. Future research should directly test this by relating individual differences in representational persistence (assessed via measures of phantom sensations or via implicit and explicit measures of prosthetic embodiment when a prosthesis is used(Zbinden et al. 2022) ) to oscillatory dynamics.

The theoretical framework outlined above should be interpreted as a hypothesis-generating proposal rather than a validated mechanistic account. Several considerations warrant caution. First, the framework rests on exploratory findings with inconsistent replication: the amputation-related PAF-Max effect did not replicate in sensitivity analyses or for PAF-CoG, and the pain intensity–PAF-CoG association was modest, measure-specific, and absent for theta power and PAF-Max. Second, our inferences about body schema integrity, representational persistence, and thalamocortical dysrhythmia are derived from spatial patterns and spectral shifts rather than direct measurement of these constructs. We did not independently assess phantom sensation phenomenology, body schema coherence, or cortical representation strength, limiting our ability to test the proposed mechanism directly. Third, although peak alpha frequency is known to decrease with age in healthy populations, we did not observe a robust age-related effect in the present data (Gil Ávila et al. 2023; Merkin et al., 2023; Park et al., 2024). This likely reflects limited statistical power and collinearity between age and amputation status in our sample and underscores the need for cautious interpretation of PAF-related findings in this cohort. Finally, the framework represents a post-hoc integration constructed to reconcile disparate findings, which carries risk of overfitting explanations to sample-specific patterns. Direct tests, including longitudinal tracking of oscillations alongside phantom phenomenology, multimodal integration of EEG with measures of representational persistence, and experimental manipulation of body schema via prosthetic embodiment or virtual reality will be critical to validate, refine, or refute this proposal.

Our findings highlight that different measures of peak alpha frequency may yield divergent results. While PAF-Max showed an amputation-related effect in the primary analysis, this effect was absent for PAF-CoG. Conversely, in exploratory analyses within the PLP group, pain intensity was positively associated with PAF-CoG but not with PAF-Max. These measures differ in their sensitivity to spectral shape and noise: PAF-Max is driven by the single frequency bin with maximal power, whereas PAF-CoG reflects the weighted center of mass across the alpha band. While PAF-Max aims to identify the true peak frequency, it doesn’t consider that there could be multiple peaks within the alpha range or that the peak could be very small in power. Additionally, even within the same metric there may exist variability in estimates based on methodological choices (Corcoran et al., 2018). Variability in the choice of peak alpha frequency metric and method of estimation may therefore contribute to inconsistent findings in the literature on pain-related alpha slowing (Dos Santos Pinheiro et al. 2016; Mussigmann et al. 2022; Zebhauser et al. 2023). Future studies should explicitly justify the selected measure and, where possible, report multiple estimates to improve comparability across studies.

The present study was powered to detect moderate to large effects, as indicated by the pre-registered effect size estimates. Post-hoc inspection of effect size estimates indicates that the amputation-related shift in PAF-Max observed in the primary mixed-effects model corresponds to a small-to-moderate partial effect (Cohen’s $\:{\mathrm{f}}^{\mathrm{2}}$=0.089). This magnitude is below the minimum effect size the present study was powered to detect with conventional power (80%), making the lack of consistent replication across sensitivity analyses and PAF-CoG unsurprising. While this effect size may be sufficient to yield statistically significant results under some analytic approaches, it is unlikely to be detected reliably in modest samples. Power calculations based on this effect size suggest that substantially larger samples (on the order of *n* ≥ 70 subjects) would be required for robust and reproducible detection. Together, these findings suggest that the observed amputation-related alpha-band effect may reflect a genuine but modest phenomenon that warrants confirmation in larger, well-powered cohorts.

Several limitations should be considered when interpreting the present findings. First, the sample size was modest and groups were unbalanced with respect to demographic and clinical characteristics, limiting statistical power and increasing uncertainty around effect estimates. Second, although mixed-effects modeling and resampling-based inference were used to mitigate these limitations, the results should be interpreted as preliminary and in need of replication in larger, better-balanced cohorts. Furthermore, scalp EEG has inherent spatial resolution limitations, and effects attributed to specific regions should be interpreted cautiously.

A critical limitation is that we did not systematically assess non-painful phantom sensations (e.g., phantom limb awareness, movement sensations, telescoping). Our theoretical framework proposes that representational persistence counteracts dysrhythmia and can be driven by both phantom sensations and pain, but we cannot test whether individuals without PLP differ in non-painful phantom experiences. This prevents us from distinguishing between ‘no phantom sensation’ and ‘phantom sensation without pain,’ limiting our ability to definitively attribute alpha changes to representation strength versus pain specifically. Additionally, prosthesis use and embodiment were not systematically assessed; prosthetic integration may also modulate the body schema.

Finally, there may exist additional influential covariates not accounted for. Time since amputation represents a potentially important moderator that should be included in future work. Similarly, amputation limb (upper or lower extremity), side (left or right) and level are all factors that may influence the spatial location and extent of alterations in cortical oscillations. These factors may be particularly relevant in studies further investigating potential biomarkers with hemispheric lateralization. We did not control for pain intensity in the main analysis. Pain intensity and cortical activity fluctuate over time, and repeated-measures designs tracking within-subject variability may be more sensitive to pain-related changes than cross-sectional group comparisons. Nor did we systematically control for analgesic medications or other concomitant treatments, antidepressants, or anticonvulsants, all of which can influence cortical oscillations. Medication effects may have added unexplained variance to our models. Incorporating more detailed measures of pain intensity, duration, or variability and considering pharmacological interventions as covariates or stratification factors, may provide greater sensitivity to pain-related neural correlates in future studies.

## Conclusions

Across all primary and sensitivity analyses the results provide no evidence for a robust association between PLP and either theta power or peak alpha frequency. An amputation-related shift in PAF-Max reached significance in the primary mixed-effects model but the effect did not replicate in the subject-level sensitivity or PAF-CoG analyses. Additional exploratory analyses support the existence of an amputation-related difference in the alpha band, but the exploratory nature of these analyses along with inconsistency in the primary analyses indicate that the findings should be interpreted with caution. In a secondary exploratory analysis restricted to amputees with PLP, pain intensity showed a small positive association with PAF-CoG. However, this effect was modest in magnitude and did not generalize to other spectral measures, and should therefore be interpreted cautiously.

## Electronic Supplementary Material

Below is the link to the electronic supplementary material.

Supplementary Material 1

## Data Availability

The data is available open access in BIDS-EEG format at: [https://openneuro.org/datasets/ds006921](https:/openneuro.org/datasets/ds006921)Code for the statistical analysis is available at: [https://doi.org/10.5281/zenodo.17977343](https:/doi.org/10.5281/zenodo.17977343).

## References

[CR1] Andoh J, Milde C, Diers M, Bekrater-Bodmann R, Trojan J, Fuchs X, Becker S, Desch S, Flor H (2020) Assessment of cortical reorganization and preserved function in phantom limb pain: a methodological perspective. Sci Rep 10(1):1–15. 10.1038/s41598-020-68206-9 32661345 10.1038/s41598-020-68206-9 PMC7359300

[CR2] Bagheri Z, Khosrowabadi R, Hatami J, Kian ARA, Fatemi MJ, Khatibi A (2023) Differential Cortical Oscillatory Patterns in Amputees With and Without Phantom Limb Pain. Basic Clin Neurosci 14(2). 10.32598/bcn.2021.261.1 10.32598/bcn.2021.261.1 PMC10719972 38107525

[CR3] Birbaumer N, Lutzenberger W, Montoya P, Larbig W, Unertl K, Töpfner S, Grodd W, Taub E, Flor H (1997) Effects of Regional Anesthesia on Phantom Limb Pain Are Mirrored in Changes in Cortical Reorganization. *The Journal of Neuroscience*, *17*(14), 5503 LP – 5508. 10.1523/JNEUROSCI.17-14-05503.1997 10.1523/JNEUROSCI.17-14-05503.1997 PMC6793813 9204932

[CR4] Bollimunta A, Chen Y, Schroeder CE, Ding M (2008) Neuronal mechanisms of cortical alpha oscillations in awake-behaving macaques. J Neurosci 28(40). 10.1523/JNEUROSCI.2699-08.2008 10.1523/JNEUROSCI.2699-08.2008 PMC2692971 18829955

[CR5] Buzsaki, G., & Draguhn, A. (2004). Neuronal Oscillations in Cortical Networks. Science, 304(5679), 1926–1929. 10.1126/science.1099745 10.1126/science.1099745 15218136

[CR6] Corcoran, A. W., Alday, P. M., Schlesewsky, M., & Bornkessel-Schlesewsky, I. (2018). Toward a reliable, automated method of individual alpha frequency (IAF) quantification. Psychophysiology, 55(7). 10.1111/psyp.13064 10.1111/psyp.13064 29357113

[CR7] Dos Santos Pinheiro ES, De Queirós FC, Montoya P, Santos CL, Nascimento D, Ito MA, Silva CH, Santos M, Benevides DBN, Miranda S, Sá JGV, K. N., Baptista AF (2016) Electroencephalographic patterns in chronic pain: A systematic review of the literature. PLoS ONE (Vol 11(2). 10.1371/journal.pone.0149085 10.1371/journal.pone.0149085 PMC4767709 26914356

[CR8] Flor H, Elbert T, Knecht S, Wienbruch C, Pantev C, Birbaumers N, Larbig W, Taub E (1995) Phantom-limb pain as a perceptual correlate of cortical reorganization following arm amputation. Nature 375(6531):482–484. 10.1038/375482a0 7777055 10.1038/375482a0

[CR9] Foell J, Bekrater-Bodmann R, Diers M, Flor H (2014) Mirror therapy for phantom limb pain: Brain changes and the role of body representation. Eur J Pain 18(5):729–739. 10.1002/j.1532-2149.2013.00433.x 24327313 10.1002/j.1532-2149.2013.00433.x

[CR10] Gil Ávila C, Bott FS, Tiemann L, Hohn VD, May ES, Nickel MM, Zebhauser PT, Gross J, Ploner M (2023) DISCOVER-EEG: an open, fully automated EEG pipeline for biomarker discovery in clinical neuroscience. Sci Data 10(1). 10.1038/s41597-023-02525-0 10.1038/s41597-023-02525-0 PMC10495446 37696851

[CR11] Grüsser SM, Winter C, Mühlnickel W, Denke C, Karl A, Villringer K, Flor H (2001) The relationship of perceptual phenomena and cortical reorganization in upper extremity amputees. Neuroscience 102(2). 10.1016/S0306-4522(00)00491-7 10.1016/s0306-4522(00)00491-7 11166112

[CR12] Haegens, S., Barczak, A., Musacchia, G., Lipton, M. L., Mehta, A. D., Lakatos, P., & Schroeder, C. E. (2015). Laminar profile and physiology of the α rhythm in primary visual, auditory, and somatosensory regions of neocortex. Journal of Neuroscience, 35(42). 10.1523/JNEUROSCI.0600-15.2015 10.1523/JNEUROSCI.0600-15.2015 PMC4683691 26490871

[CR13] Heilman KM, Valenstein E, Watson RT (2000) Neglect and related disorders. In *Seminars in Neurology* (Vol. 20, Issue 4). 10.1055/s-2000-13179 10.1055/s-2000-13179 11149702

[CR14] Hughes SW, Crunelli V (2005) Thalamic mechanisms of EEG alpha rhythms and their pathological implications. In *Neuroscientist* (Vol. 11, Issue 4). 10.1177/1073858405277450 10.1177/1073858405277450 16061522

[CR15] Huse E, Larbig W, Flor H, Birbaumer N (2001) The effect of opioids on phantom limb pain and cortical reorganization. Pain 90:1–2. 10.1016/S0304-3959(00)00385-7 11166969 10.1016/s0304-3959(00)00385-7

[CR16] Karl A, Birbaumer N, Lutzenberger W, Cohen LG, Flor H (2001) Reorganization of Motor and Somatosensory Cortex in Upper Extremity Amputees with Phantom Limb Pain. J Neurosci 21(10) 3609 LP – 3618. 10.1523/JNEUROSCI.21-10-03609.2001 10.1523/JNEUROSCI.21-10-03609.2001 PMC6762494 11331390

[CR17] Karnath HO, Ferber S, Himmelbach M (2001) Spatial awareness is a function of the temporal not the posterior parietal lobe. Nature 411(6840). 10.1038/35082075 10.1038/35082075 11418859

[CR18] Kikkert S, Johansen-Berg H, Tracey I, Makin TR (2018) Reaffirming the link between chronic phantom limb pain and maintained missing hand representation. Cortex 106:174–184. 10.1016/j.cortex.2018.05.013 30005369 10.1016/j.cortex.2018.05.013 PMC6143485

[CR19] Klug M, Kloosterman NA (2022) Zapline-plus: A Zapline extension for automatic and adaptive removal of frequency-specific noise artifacts in M/EEG. Hum Brain Mapp 43(9). 10.1002/hbm.25832 10.1002/hbm.25832 PMC9120550 35278015

[CR20] Knecht S, Henningsen H, Elbert T, Flor H, Höhling C, Pantev C, Birbaumer N, Taub E (1995) Cortical reorganization in human amputees and mislocalization of painful stimuli to the phantom limb. Neurosci Lett 201(3). 10.1016/0304-3940(95)12186-2 10.1016/0304-3940(95)12186-2 8786855

[CR21] Lendaro E, Van der Sluis CK, Hermansson L, Bunketorp-Käll L, Burger H, Keesom E, Widehammar C, Munoz-Novoa M, McGuire BE, O’Reilly P, Earley EJ, Iqbal S, Kristoffersen MB, Stockselius A, Gudmundson L, Hill W, Diers M, Turner KL, Weiss T, Ortiz-Catalan M (2025) Extended reality used in the treatment of phantom limb pain: a multicenter, double-blind, randomized controlled trial. Pain 166(3):571–586. 10.1097/j.pain.0000000000003384 39250328 10.1097/j.pain.0000000000003384 PMC11808706

[CR22] Limakatso K, Bedwell GJ, Madden VJ, Parker R (2020) The prevalence and risk factors for phantom limb pain in people with amputations: A systematic review and meta-analysis. PLoS ONE 15(10):e0240431. 10.1371/journal.pone.0240431 33052924 10.1371/journal.pone.0240431 PMC7556495

[CR23] Llinás RR, Ribary U, Jeanmonod D, Kronberg E, Mitra PP (1999) Thalamocortical dysrhythmia: A neurological and neuropsychiatric syndrome characterized by magnetoencephalography. Proc Natl Acad Sci USA 96(26). 10.1073/pnas.96.26.15222 10.1073/pnas.96.26.15222 PMC24801 10611366

[CR24] Lopes da Silva FH, Vos JE, Mooibroek J, van Rotterdam A (1980) Relative contributions of intracortical and thalamo-cortical processes in the generation of alpha rhythms, revealed by partial coherence analysis. Electroencephalogr Clin Neurophysiol 50(5–6). 10.1016/0013-4694(80)90011-5 10.1016/0013-4694(80)90011-5 6160987

[CR25] Lotze M, Flor H, Grodd W, Larbig W, Birbaumer N (2001) Phantom movements and pain An fMRI study in upper limb amputees. Brain 124(11):2268–2277. 10.1093/brain/124.11.2268 11673327 10.1093/brain/124.11.2268

[CR26] MacIver K, Lloyd DM, Kelly S, Roberts N, Nurmikko T (2008) Phantom limb pain, cortical reorganization and the therapeutic effect of mental imagery. Brain 131(8). 10.1093/brain/awn124 10.1093/brain/awn124 PMC2494616 18567624

[CR27] Makin TR, Filippini N, Duff EP, Henderson Slater D, Tracey I, Johansen-Berg H (2015a) Network-level reorganisation of functional connectivity following arm amputation. NeuroImage 114:217–225. 10.1016/j.neuroimage.2015.02.067 25776216 10.1016/j.neuroimage.2015.02.067 PMC4461307

[CR28] Makin TR, Scholz J, Filippini N, Slater H, Tracey D, I., Johansen-Berg H (2013) Phantom pain is associated with preserved structure and function in the former hand area. Nat Commun 4(1):1570. 10.1038/ncomms2571 23463013 10.1038/ncomms2571 PMC3615341

[CR29] Makin TR, Scholz J, Henderson Slater D, Johansen-Berg H, Tracey I (2015b) Reassessing cortical reorganization in the primary sensorimotor cortex following arm amputation. Brain 138(8):2140–2146. 10.1093/brain/awv161 26072517 10.1093/brain/awv161 PMC4511862

[CR30] Merkin, A., Sghirripa, S., Graetz, L., Smith, A. E., Hordacre, B., Harris, R., Pitcher, J., Semmler, J., Rogasch, N. C., & Goldsworthy, M. (2023). Do age-related differences in aperiodic neural activity explain differences in resting EEG alpha? Neurobiology of Aging, 121. 10.1016/j.neurobiolaging.2022.09.003 10.1016/j.neurobiolaging.2022.09.003 36379095

[CR31] Mussigmann T, Bardel B, Lefaucheur JP (2022) Resting-state electroencephalography (EEG) biomarkers of chronic neuropathic pain. A systematic review. In *NeuroImage* (Vol. 258). 10.1016/j.neuroimage.2022.119351 10.1016/j.neuroimage.2022.119351 35659993

[CR32] Oostenveld R, Fries P, Maris E, Schoffelen JM (2011) FieldTrip: Open source software for advanced analysis of MEG, EEG, and invasive electrophysiological data. Comput Intell Neurosci 2011. 10.1155/2011/156869 10.1155/2011/156869 PMC3021840 21253357

[CR33] Park, J., Ho, R. L. M., Wang, W. en, Nguyen, V. Q., & Coombes, S. A. (2024). The effect of age on alpha rhythms in the human brain derived from source localized resting-state electroencephalography. NeuroImage, 292. 10.1016/j.neuroimage.2024.120614 10.1016/j.neuroimage.2024.120614 38631618

[CR34] Pfurtscheller G, Stancák A, Neuper C (1996) Event-related synchronization (ERS) in the alpha band - An electrophysiological correlate of cortical idling: A review. Int J Psychophysiol 24(1–2). 10.1016/S0167-8760(96)00066-9 10.1016/s0167-8760(96)00066-9 8978434

[CR35] Ploner M, Sorg C, Gross J (2017) Brain Rhythms of Pain. In *Trends in Cognitive Sciences* (Vol. 21, Issue 2). 10.1016/j.tics.2016.12.001 10.1016/j.tics.2016.12.001 PMC5374269 28025007

[CR36] Raffin E, Richard N, Giraux P, Reilly KT (2016) Primary motor cortex changes after amputation correlate with phantom limb pain and the ability to move the phantom limb. NeuroImage 130:134–144. 10.1016/j.neuroimage.2016.01.063 26854561 10.1016/j.neuroimage.2016.01.063

[CR37] Schone HR, Baker CI, Katz J, Nikolajsen L, Limakatso K, Flor H, Makin TR (2022) Making sense of phantom limb pain. *Journal of Neurology, Neurosurgery & Psychiatry*, *93*(8), 833 LP – 843. 10.1136/jnnp-2021-328428 10.1136/jnnp-2021-328428 PMC9304093 35609964

[CR38] Vallar G, Perani D (1986) The anatomy of unilateral neglect after right-hemisphere stroke lesions. A clinical/CT-scan correlation study in man. Neuropsychologia 24(5). 10.1016/0028-3932(86)90001-1 10.1016/0028-3932(86)90001-1 3785649

[CR39] Zbinden J, Lendaro E, Ortiz-Catalan M (2022) Prosthetic embodiment: systematic review on definitions, measures, and experimental paradigms. In *Journal of NeuroEngineering and Rehabilitation* (Vol. 19, Issue 1). 10.1186/s12984-022-01006-6 10.1186/s12984-022-01006-6 PMC8962549 35346251

[CR40] Zebhauser PT, Hohn VD, Ploner M (2023) Resting-state electroencephalography and magnetoencephalography as biomarkers of chronic pain: A systematic review. Pain 164(6). 10.1097/j.pain.0000000000002825 10.1097/j.pain.0000000000002825 PMC10184564 36409624

