# Supplementary material for: Resting-State Theta and Alpha Oscillations in Amputation and Phantom Limb Pain: A Pre-Registered High-Density EEG Study
Source: Brain Topogr. 2026 May 22;39(4):58. doi: 10.1007/s10548-026-01210-w (PMC13197317; doi:10.1007/s10548-026-01210-w)
Supplement: Supplementary file 1 — Supplementary Material 1 [file 10548_2026_1210_MOESM1_ESM.docx]

Supplemental material

Supporting the manuscript A Cross-Sectional Analysis of Neuro-Oscillatory Correlates of Phantom Limb Pain and Amputation: A Resting State High-Density EEG Study

Table of Contents

[Model fit and diagnostics 1](#_Toc216886391)

[Research Question 1: Theta Power 1](#_Toc216886392)

[Linear mixed-effects model of theta power 1](#_Toc216886393)

[Subject-level linear model of theta power 3](#_Toc216886394)

[Research Question 2: Peak Alpha Frequency 4](#_Toc216886395)

[Linear mixed-effects model of PAF-Max 4](#_Toc216886396)

[Subject-level linear model of PAF-Max 6](#_Toc216886397)

[Linear mixed-effects model of PAF-CoG 8](#_Toc216886398)

[Subject-level linear model of PAF-CoG 9](#_Toc216886399)

[Effect size estimation 11](#_Toc216886400)

# Model fit and diagnostics

## Research Question 1: Theta Power

### Linear mixed-effects model of theta power


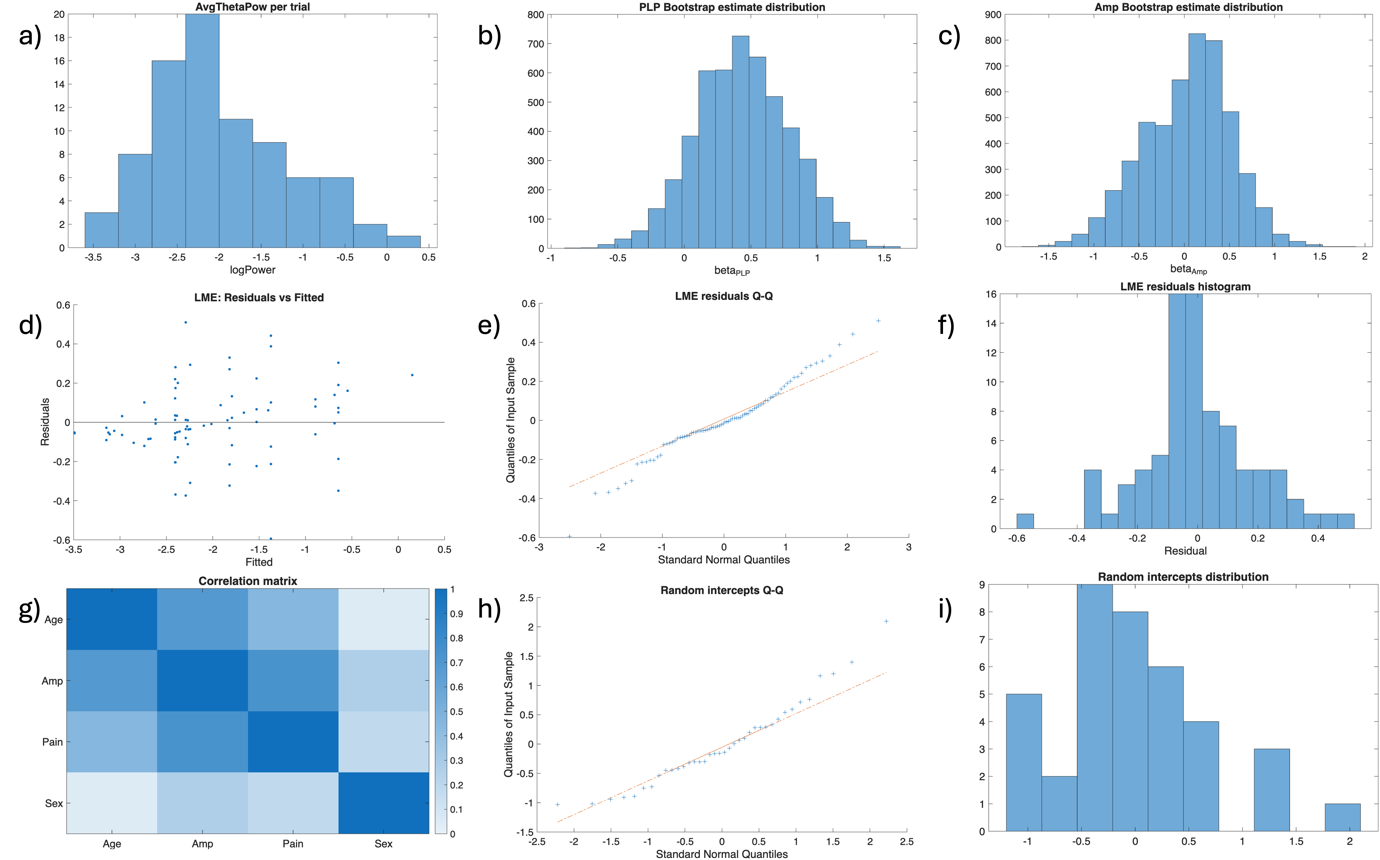


Figure S 1 Diagnostics for the linear mixed-effects model of whole-brain theta power. (a) Histogram of log-transformed average theta power per trial. (b) Distribution of the estimated PLP fixed-effect coefficient obtained from 5,000 subject-level bootstrap resamples. (c) Distribution of the estimated Amputation fixed-effect coefficient obtained from 5,000 subject-level bootstrap resamples. (d) Residuals plotted against fitted values from the linear mixed-effects model. (e) Quantile–quantile (Q–Q) plot of model residuals. (f) Histogram of model residuals. (g) Pairwise correlation matrix of fixed-effect predictors included in the model. (h) Q–Q plot of subject-specific random intercepts. (i) Histogram of subject-specific random intercepts.

| **Model fit statistic** | **Value** |
| --- | --- |
| Marginal R^2^ | 0.271 |
| Conditional R^2^ | 0.883 |
| VIF Age | 1.9423 |
| VIF Amp | 3.4419 |
| VIF Pain | 2.1707 |
| VIF Sex | 1.1105 |

Model fit was quantified using marginal and conditional R² values. The marginal R² reflects the variance explained by the fixed effects alone, while the conditional R² reflects the variance explained by both fixed and random effects. The difference between these values indicates that a substantial portion of variance was attributable to between-subject differences captured by the random intercepts.

The histogram of the dependent variable (Fig S1 a) indicate a somewhat skewed distribution. No extreme values suggesting gross violations of distributional assumptions are observed.

The bootstrap distributions of the fixed-effect coefficients for PLP and Amputation (Fig S1 b and c) are approximately bell-shaped, with slight skewness. The absence of multimodality suggests that estimates were not driven by a small number of influential subjects.

Residuals (Fig S1 d) are centered around zero across the range of fitted values, with no clear systematic structure, providing no strong evidence for heteroscedasticity or major model misspecification. The Q–Q plot and histogram (Fig S1 e and f) show some deviations from normality, primarily in the end ranges. Given the limited sample size, these deviations were considered acceptable and were further accounted for through the use of bootstrap-based confidence intervals.

Pairwise correlations among fixed-effect predictors (Fig S1 g) revealed strong association between Pain and Amputee status (r ≈ 0.7 across trials), reflecting the nested structure of the sample, and a moderate-to-high correlation between Age and Amputee (r ≈ 0.8), consistent with demographic imbalance between groups. Variance inflation factors (VIFs) for all predictors were below the conventional threshold of (5), indicating that multicollinearity was unlikely to substantially inflate standard errors or compromise interpretability of the fixed-effect estimates.

The Q–Q plot and histogram of subject-specific random intercepts (Fig S1 h and i) showed some deviations from normality. No extreme outliers were observed, supporting the adequacy of the random-intercept specification.

The model converged without warnings. **Taken together, the model diagnostics did not indicate violations severe enough to invalidate the resulting inferences.**

### Subject-level linear model of theta power


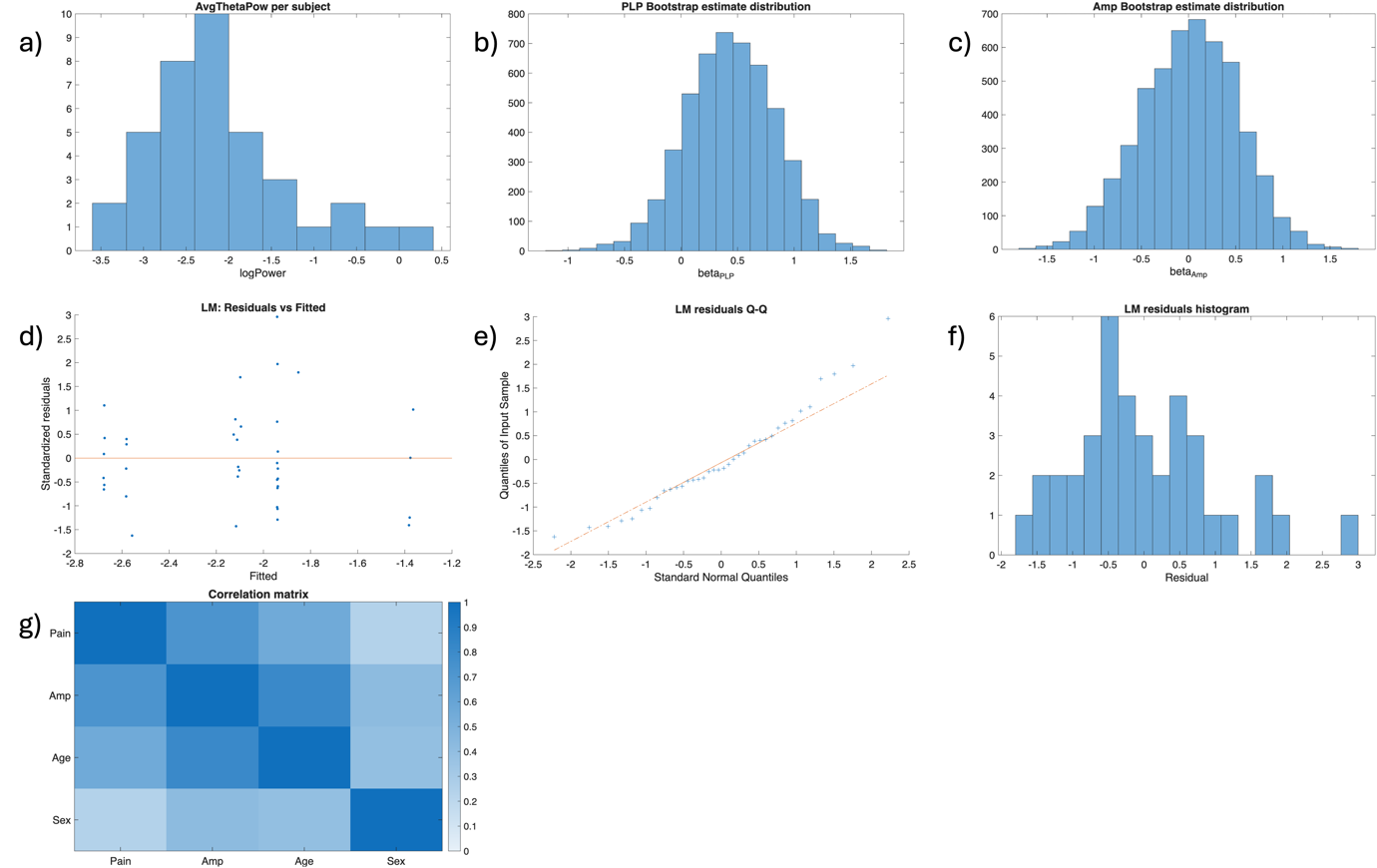


Figure S 2 Diagnostics for the subject level linear model of whole-brain theta power. (a) Histogram of log-transformed average theta power per subject. (b) Distribution of the estimated PLP fixed-effect coefficient obtained from 5,000 subject-level bootstrap resamples. (c) Distribution of the estimated Amputation fixed-effect coefficient obtained from 5,000 subject-level bootstrap resamples. (d) Residuals plotted against fitted values from the linear model. (e) Quantile–quantile (Q–Q) plot of model residuals. (f) Histogram of model residuals. (g) Pairwise correlation matrix of predictors included in the model.

| **Model fit statistic** | **Value** |
| --- | --- |
| Ordinary R^2^ | 0.203 |
| Adjusted R^2^ | 0.106 |
| VIF Age | 2.915 |
| VIF Amp | 4.3999 |
| VIF Pain | 2.1145 |
| VIF Sex | 1.2375 |

Model fit was quantified using the adjusted R² value, as it accounts for the number of predictors relative to sample size. The adjusted R² indicates that the model explained a moderate proportion (20%) of variance in the dependent variable. For completeness, the ordinary R² is also reported, reflecting the proportion of variance explained by the predictors without correction for model complexity

The histogram of the dependent variable (Fig S2 a) indicate a somewhat skewed distribution. No extreme values suggesting gross violations of distributional assumptions are observed.

The bootstrap distributions of the fixed-effect coefficients for PLP and Amputation (Fig S2 b and c) are approximately bell-shaped. The absence of multimodality suggests that estimates were not driven by a small number of influential subjects.
Residuals (Fig S2 d) are centered around zero across the range of fitted values, with no clear systematic structure, providing no strong evidence for heteroscedasticity or major model misspecification. The Q–Q plot and histogram (Fig S2 e and f) show some deviations from normality, primarily in the end ranges. Given the limited sample size, these deviations were considered acceptable and were further accounted for through the use of bootstrap-based confidence intervals.

Pairwise correlations among predictors (Fig S2 g) revealed strong association between Pain and Amputee status (r ≈ 0.7 across trials), reflecting the nested structure of the sample, and a high correlation between Age and Amputee (r ≈ 0.9), consistent with demographic imbalance between groups. Variance inflation factors (VIFs) for all predictors were below the conventional threshold of (5), indicating that multicollinearity was unlikely to substantially inflate standard errors or compromise interpretability of the fixed-effect estimates.

The model converged without warnings. **Taken together, the model diagnostics did not indicate violations severe enough to invalidate the resulting inferences.**

## Research Question 2: Peak Alpha Frequency

### Linear mixed-effects model of PAF-Max


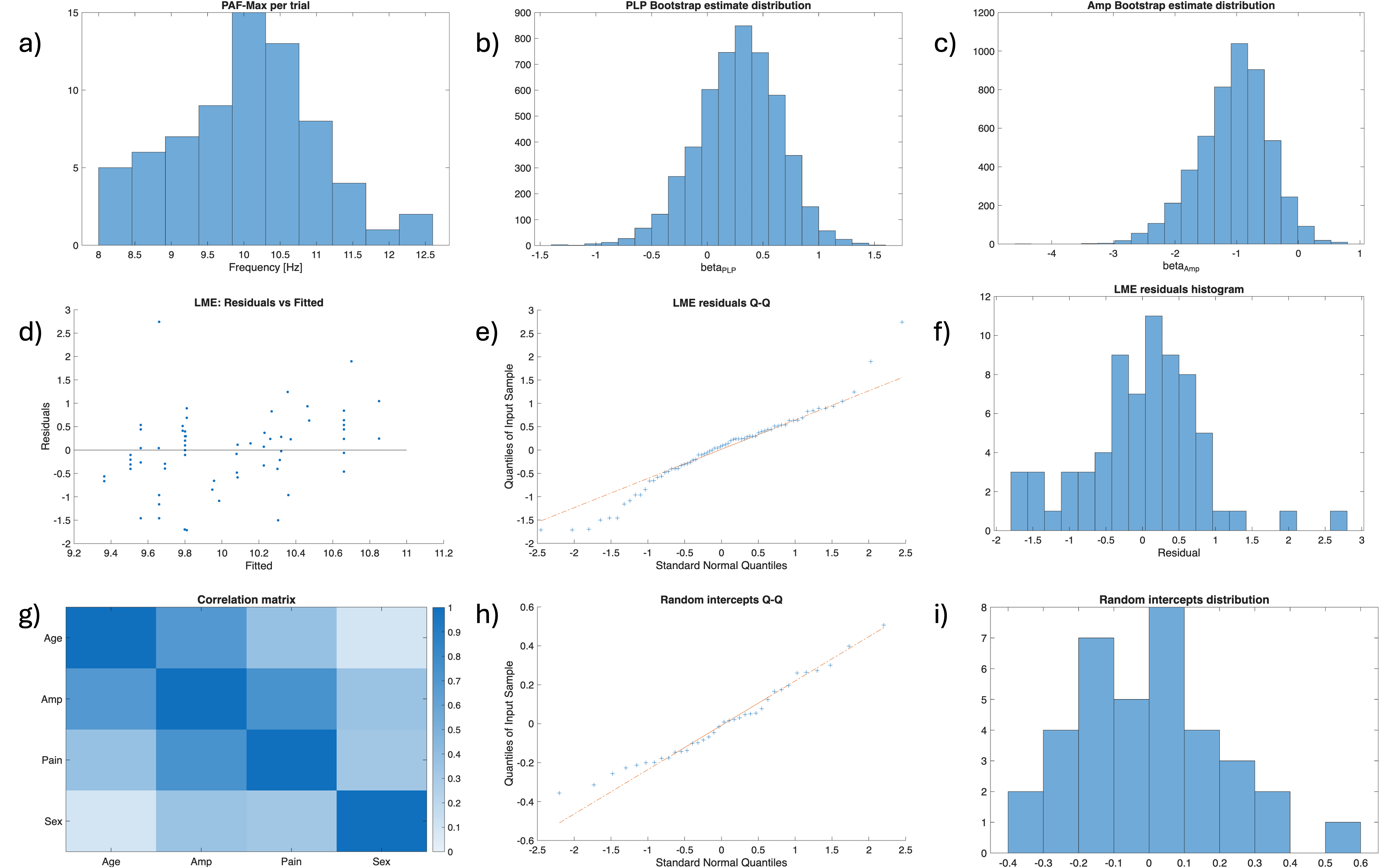


Figure S 3 Diagnostics for the linear mixed-effects model of whole-brain PAF-Max. (a) Histogram of PAF-Max per trial. (b) Distribution of the estimated PLP fixed-effect coefficient obtained from 5,000 subject-level bootstrap resamples. (c) Distribution of the estimated Amputation fixed-effect coefficient obtained from 5,000 subject-level bootstrap resamples. (d) Residuals plotted against fitted values from the linear mixed-effects model. (e) Quantile–quantile (Q–Q) plot of model residuals. (f) Histogram of model residuals. (g) Pairwise correlation matrix of fixed-effect predictors included in the model. (h) Q–Q plot of subject-specific random intercepts. (i) Histogram of subject-specific random intercepts.

| **Model fit statistic** | **Value** |
| --- | --- |
| Marginal R^2^ | 0.082 |
| Conditional R^2^ | 0.164 |
| VIF Age | 2.1753 |
| VIF Amp | 4.2906 |
| VIF Pain | 2.388 |
| VIF Sex | 1.2244 |

Model fit was quantified using marginal and conditional R² values. The marginal R² reflects the variance explained by the fixed effects alone, while the conditional R² reflects the variance explained by both fixed and random effects. For PAF-Max these values indiacte that the mixed-effects model explained only a modest proportion of variance.

The histogram of the dependent variable (Fig S3 a) indicate a somewhat skewed distribution. No extreme values suggesting gross violations of distributional assumptions are observed.

The bootstrap distributions of the fixed-effect coefficients for PLP and Amputation (Fig S3 b and c) are approximately bell-shaped, with slight skewness. The absence of multimodality suggests that estimates were not driven by a small number of influential subjects.

Residuals (Fig S3 d) are centered around zero across the range of fitted values, with no clear systematic structure, providing no strong evidence for heteroscedasticity or major model misspecification. The Q–Q plot and histogram (Fig S3 e and f) show some deviations from normality, primarily in the end ranges. Given the limited sample size, these deviations were considered acceptable and were further accounted for through the use of bootstrap-based confidence intervals.

Pairwise correlations among fixed-effect predictors (Fig S3 g) revealed strong association between Pain and Amputee status (r ≈ 0.7 across trials), reflecting the nested structure of the sample, and a moderate-to-high correlation between Age and Amputee (r ≈ 0.8), consistent with demographic imbalance between groups. Variance inflation factors (VIFs) for all predictors were below the conventional threshold of (5), indicating that multicollinearity was unlikely to substantially inflate standard errors or compromise interpretability of the fixed-effect estimates.

The Q–Q plot and histogram of subject-specific random intercepts (Fig S3 h and i) showed some deviations from normality. No extreme outliers were observed, supporting the adequacy of the random-intercept specification.

The model converged without warnings. **Taken together, the model diagnostics indicate that results should be interpreted with appropriate caution given the modest sample size, which limits the precision of fixed-effect estimates and results in low explained variance and some deviations from normality assumptions. These issues are partially mitigated through the use of resampling-based inference.**

### Subject-level linear model of PAF-Max


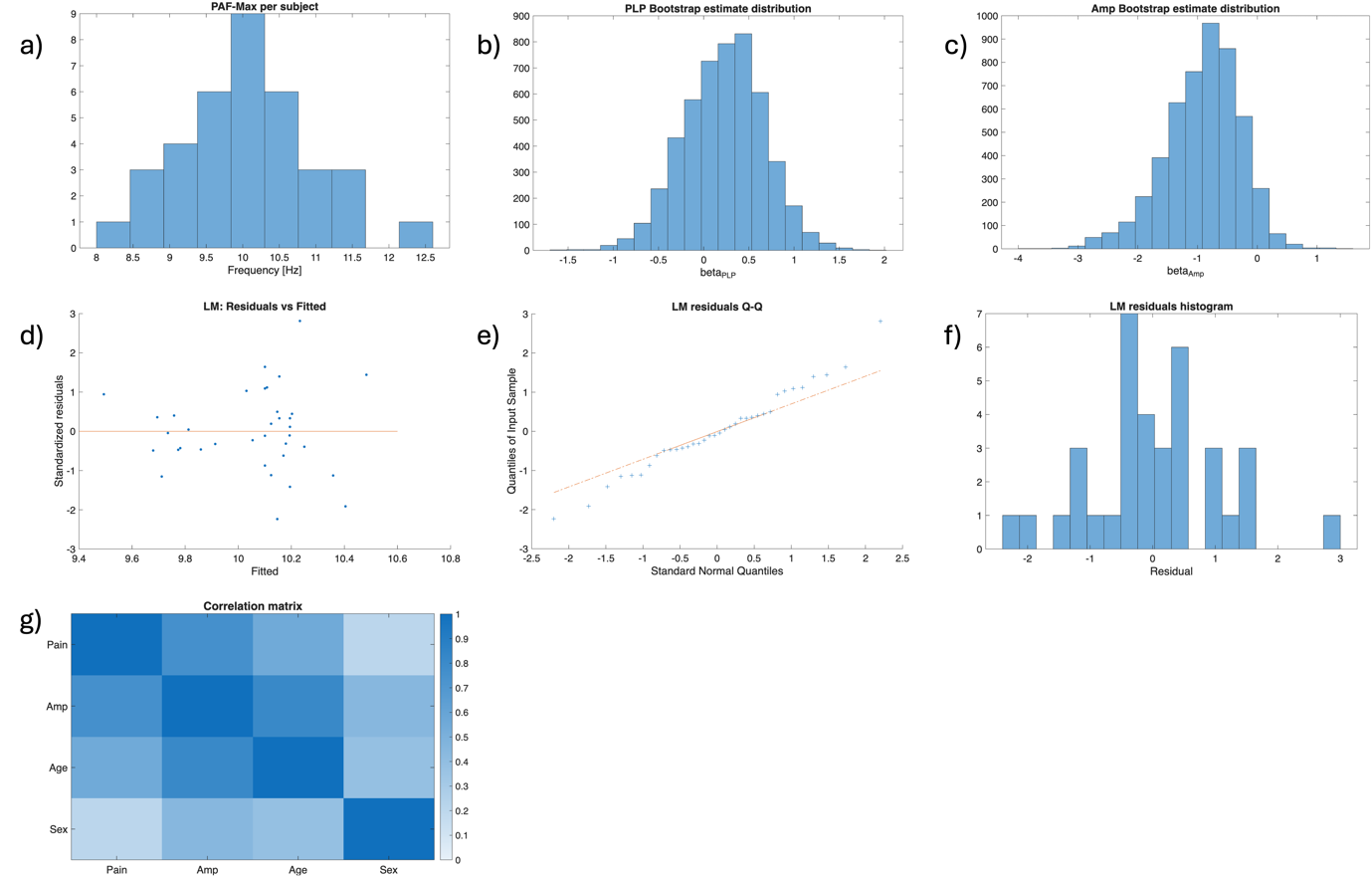


Figure S 4 Diagnostics for the subject level linear model of whole-brain PAF-Max. (a) Histogram of PAF-Max per subject. (b) Distribution of the estimated PLP fixed-effect coefficient obtained from 5,000 subject-level bootstrap resamples. (c) Distribution of the estimated Amputation fixed-effect coefficient obtained from 5,000 subject-level bootstrap resamples. (d) Residuals plotted against fitted values from the linear model. (e) Quantile–quantile (Q–Q) plot of model residuals. (f) Histogram of model residuals. (g) Pairwise correlation matrix of predictors included in the model.

| **Model fit statistic** | **Value** |
| --- | --- |
| Ordinary R^2^ | 0.061 |
| Adjusted R^2^ | -0.060 |
| VIF Age | 2.9788 |
| VIF Amp | 5.3548 |
| VIF Pain | 2.469 |
| VIF Sex | 1.3131 |

Model fit was quantified using the adjusted R² value, as it accounts for the number of predictors relative to sample size. The low negative adjusted R² indicates that, after accounting for model complexity, the predictors did not explain substantial variance beyond that expected by chance.

The histogram of the dependent variable (Fig S4 a) indicate a somewhat skewed distribution. No extreme values suggesting gross violations of distributional assumptions are observed.

The bootstrap distributions of the fixed-effect coefficients for PLP and Amputation (Fig S4 b and c) are approximately bell-shaped. The absence of multimodality suggests that estimates were not driven by a small number of influential subjects.

Residuals (Fig S4 d) are centered around zero across the range of fitted values, with no clear systematic structure, providing no strong evidence for heteroscedasticity or major model misspecification. The Q–Q plot and histogram (Fig S4 e and f) show some deviations from normality, primarily in the end ranges. Given the limited sample size, these deviations were considered acceptable and were further accounted for through the use of bootstrap-based confidence intervals.

Pairwise correlations among predictors (Fig S4 g) revealed strong association between Pain and Amputee status (r ≈ 0.7 across trials), reflecting the nested structure of the sample, and a high correlation between Age and Amputee (r ≈ 0.9), consistent with demographic imbalance between groups. Variance inflation factors (VIFs) for all predictors were below the conventional threshold of (5) except for Amputation.

As a diagnostic (not pre-registered) test we refit the model without Amputation as a covariate, to inspect if collinearity (indicated by the high correlation and VIF Amp > 5) of Amputation and Age might be obscuring the expected Age-related effect. However, this did not strengthen the Age effect—instead, the Age coefficient became smaller (0.099) and nonsignificant (p = 0.545)—indicating that the null Age finding likely is not attributable to collinearity with Amputation.

The model converged without warnings. **Taken together, the model diagnostics indicate that results should be interpreted with appropriate caution and are presented as a robustness analysis rather than a primary inferential test.**

### Linear mixed-effects model of PAF-CoG


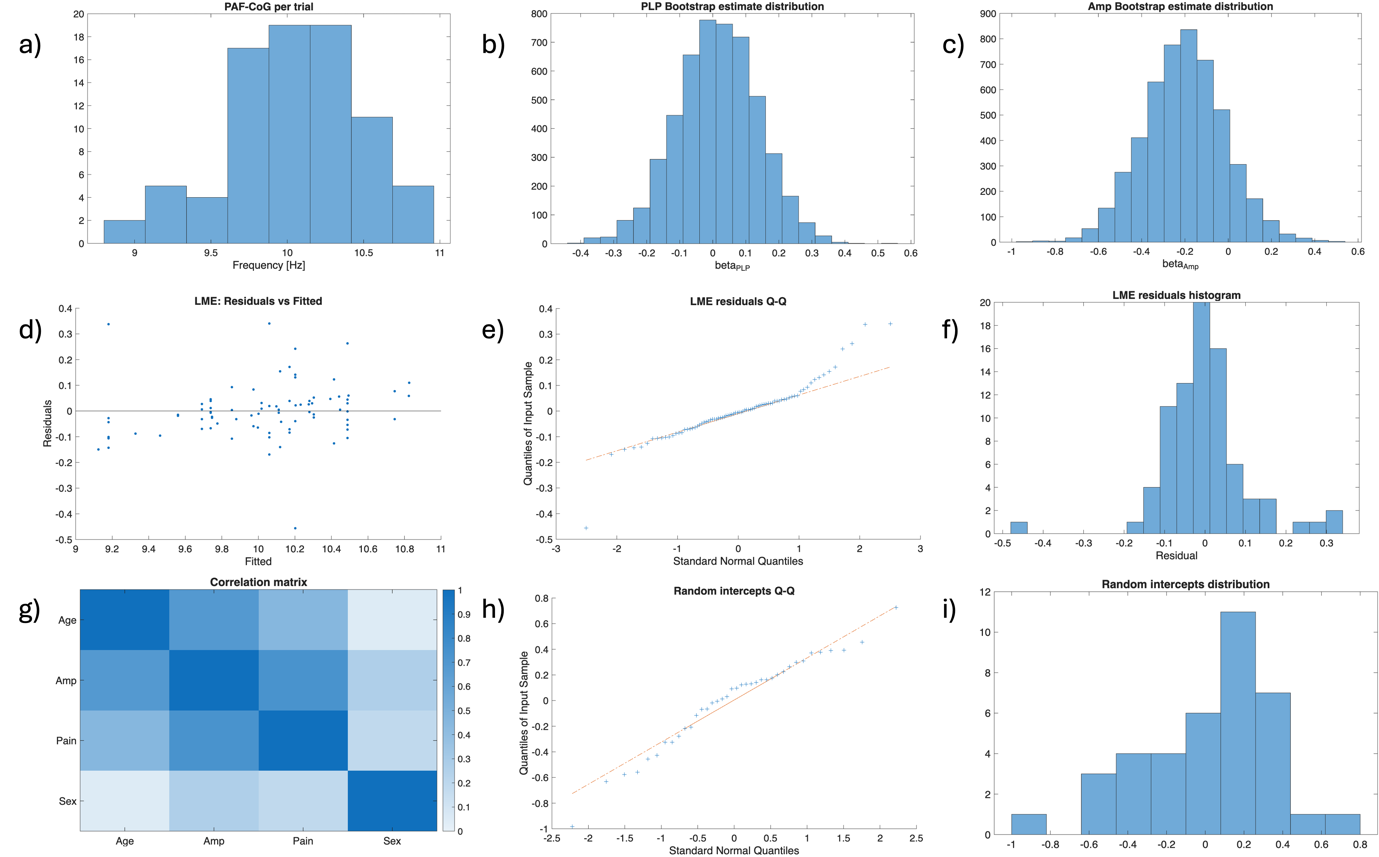


Figure S 5 Diagnostics for the linear mixed-effects model of whole-brain PAF-CoG. (a) Histogram of PAF-CoG per trial. (b) Distribution of the estimated PLP fixed-effect coefficient obtained from 5,000 subject-level bootstrap resamples. (c) Distribution of the estimated Amputation fixed-effect coefficient obtained from 5,000 subject-level bootstrap resamples. (d) Residuals plotted against fitted values from the linear mixed-effects model. (e) Quantile–quantile (Q–Q) plot of model residuals. (f) Histogram of model residuals. (g) Pairwise correlation matrix of fixed-effect predictors included in the model. (h) Q–Q plot of subject-specific random intercepts. (i) Histogram of subject-specific random intercepts.

| **Model fit statistic** | **Value** |
| --- | --- |
| Marginal R^2^ | 0.236 |
| Conditional R^2^ | 0.861 |
| VIF Age | 1.9423 |
| VIF Amp | 3.4419 |
| VIF Pain | 2.1707 |
| VIF Sex | 1.1105 |

Model fit was quantified using marginal and conditional R² values. The marginal R² reflects the variance explained by the fixed effects alone, while the conditional R² reflects the variance explained by both fixed and random effects. The difference between these values indicates that a substantial portion of variance was attributable to between-subject differences captured by the random intercepts.

The histogram of the dependent variable (Fig S5 a) indicate a somewhat skewed distribution. No extreme values suggesting gross violations of distributional assumptions are observed.

The bootstrap distributions of the fixed-effect coefficients for PLP and Amputation (Fig S5 b and c) are approximately bell-shaped. The absence of multimodality suggests that estimates were not driven by a small number of influential subjects.

Residuals (Fig S5 d) are centered around zero across the range of fitted values, with no clear systematic structure, providing no strong evidence for heteroscedasticity or major model misspecification. The Q–Q plot and histogram (Fig S5 e and f) show some deviations from normality, primarily in the end ranges. Given the limited sample size, these deviations were considered acceptable and were further accounted for through the use of bootstrap-based confidence intervals.

Pairwise correlations among fixed-effect predictors (Fig S5 g) revealed strong association between Pain and Amputee status (r ≈ 0.7 across trials), reflecting the nested structure of the sample, and a moderate-to-high correlation between Age and Amputee (r ≈ 0.8), consistent with demographic imbalance between groups. Variance inflation factors (VIFs) for all predictors were below the conventional threshold of (5), indicating that multicollinearity was unlikely to substantially inflate standard errors or compromise interpretability of the fixed-effect estimates.

The Q–Q plot and histogram of subject-specific random intercepts (Fig S5 h and i) showed some deviations from normality. No extreme outliers were observed, supporting the adequacy of the random-intercept specification.

The model converged without warnings. **Taken together, the model diagnostics did not indicate violations severe enough to invalidate the resulting inferences.**

### Subject-level linear model of PAF-CoG


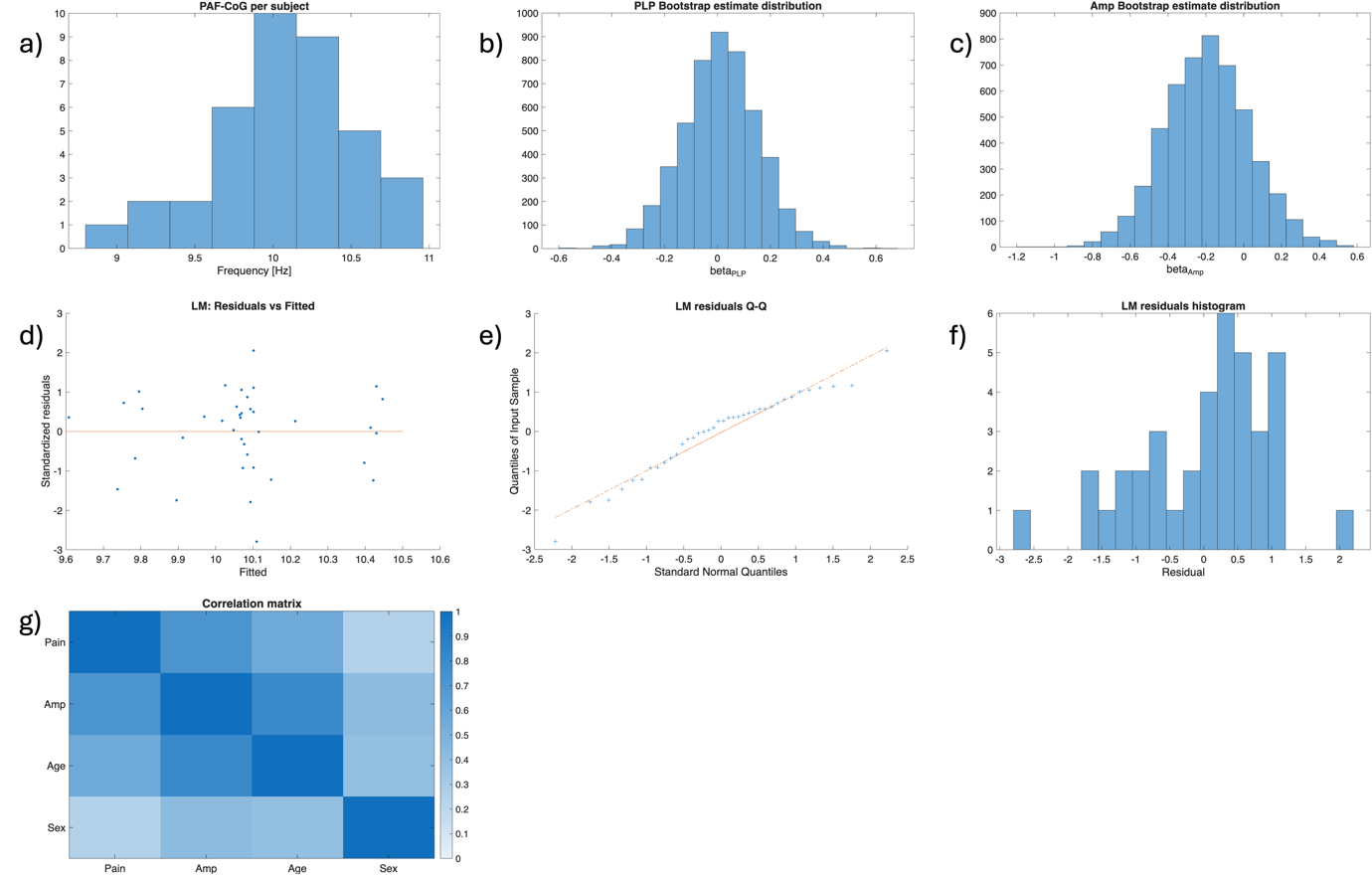


Figure S 6 Diagnostics for the subject level linear model of whole-brain PAF-CoG. (a) Histogram of PAF-CoG per subject. (b) Distribution of the estimated PLP fixed-effect coefficient obtained from 5,000 subject-level bootstrap resamples. (c) Distribution of the estimated Amputation fixed-effect coefficient obtained from 5,000 subject-level bootstrap resamples. (d) Residuals plotted against fitted values from the linear model. (e) Quantile–quantile (Q–Q) plot of model residuals. (f) Histogram of model residuals. (g) Pairwise correlation matrix of predictors included in the model.

| **Model fit statistic** | **Value** |
| --- | --- |
| Ordinary R^2^ | 0.205 |
| Adjusted R^2^ | 0.109 |
| VIF Age | 2.915 |
| VIF Amp | 4.3999 |
| VIF Pain | 2.1145 |
| VIF Sex | 1.2375 |

Model fit was quantified using the adjusted R² value, as it accounts for the number of predictors relative to sample size. The adjusted R² indicates that the model explained a moderate proportion (20%) of variance in the dependent variable. For completeness, the ordinary R² is also reported, reflecting the proportion of variance explained by the predictors without correction for model complexity

The histogram of the dependent variable (Fig S6 a) indicate a somewhat skewed distribution. No extreme values suggesting gross violations of distributional assumptions are observed.

The bootstrap distributions of the fixed-effect coefficients for PLP and Amputation (Fig S6 b and c) are approximately bell-shaped. The absence of multimodality suggests that estimates were not driven by a small number of influential subjects.

Residuals (Fig S6 d) are centered around zero across the range of fitted values, with no clear systematic structure, providing no strong evidence for heteroscedasticity or major model misspecification. The Q–Q plot and histogram (Fig S6 e and f) show some deviations from normality, primarily in the end ranges. Given the limited sample size, these deviations were considered acceptable and were further accounted for through the use of bootstrap-based confidence intervals.

Pairwise correlations among predictors (Fig S6 g) revealed strong association between Pain and Amputee status (r ≈ 0.7 across trials), reflecting the nested structure of the sample, and a high correlation between Age and Amputee (r ≈ 0.9), consistent with demographic imbalance between groups. Variance inflation factors (VIFs) for all predictors were below the conventional threshold of (5), indicating that multicollinearity was unlikely to substantially inflate standard errors or compromise interpretability of the fixed-effect estimates.

The model converged without warnings. **Taken together, the model diagnostics did not indicate violations severe enough to invalidate the resulting inferences.**

# Effect size estimation

For comparability with prior literature, marginal R² from the mixed-effects models and adjusted R² from the subject-level linear models were converted to Cohen’s f² and approximate Cohen’s d.

Conversion $\text{R}^{\text{2}} \text{f}^{\text{2}}$ according to the definition of $\text{f}^{\text{2}}$:

$$\text{f}^{\text{2}}=\frac{\text{R}^{\text{2}}}{1-\text{R}^{\text{2}}}$$

Approximate conversion $\text{R}^{\text{2}} \text{d}$ according to *Chapter 7 Converting among effect sizes* in the book *Introduction to Meta-Analysis* by Michael Borenstein, Larry V. Hedges, Julian P. T. Higgins & Hannah R. Rothstein (DOI: 10.1002/9780470743386):

$$\text{d} = \frac{2R}{\sqrt{1-\text{R}^{\text{2}}}}$$

or equivalently

$$\text{d} = 2\sqrt{\text{f}^{\text{2}}}$$
